# Acetazolamide inhibition of carbonic anhydrase 4 reverses opioid-induced synaptic rearrangements in nucleus accumbens and reduces drug-seeking behavior

**DOI:** 10.1038/s41386-025-02319-5

**Published:** 2026-01-21

**Authors:** Subhash C. Gupta, Rebecca J. Taugher-Hebl, Ali Ghobbeh, Marshal T. Jahnke, Rong Fan, Ryan T. LaLumiere, John A. Wemmie

**Affiliations:** 1 Department of Psychiatry, University of Iowa, Iowa City, IA USA; 2 Department of Veterans Affairs Medical Center, Iowa City, IA USA; 3 Interdisciplinary Graduate Program in Neuroscience, University of Iowa, Iowa City, IA USA; 4 Department of Psychological and Brain Sciences, University of Iowa, Iowa City, IA USA; 5 Iowa Neuroscience Institute, University of Iowa, Iowa City, IA USA; 6 Department of Molecular Physiology and Biophysics, University of Iowa, Iowa City, IA USA; 7 Medical Scientist Training Program, University of Iowa, Iowa City, IA USA; 8 Department of Neurosurgery, University of Iowa, Iowa City, IA USA

**Keywords:** Addiction, Reward, Motivation, Addiction

## Abstract

Persistent vulnerability to drug-seeking is driven by enduring synaptic adaptations, yet current μ-opioid receptor-targeting pharmacotherapies provide limited efficacy against these neuroadaptations. Thus, there is a critical need for mechanistically distinct, non-opioid interventions. We recently found that carbonic anhydrase 4 (CA4) disruption reduces cocaine-induced synaptic adaptations and drug-seeking. Building on this foundation, we sought to determine whether deleting CA4 or pharmacological inhibition with acetazolamide (AZD), a clinically employed carbonic anhydrase inhibitor—could mitigate opioid withdrawal–associated plasticity and thus might reduce relapse vulnerability. We studied synaptic and behavioral adaptations to withdrawal from oxycodone in mice and found that prolonged withdrawal from oxycodone increased the AMPAR/NMDAR ratio and promoted synaptic incorporation of Ca^2+^-permeable AMPARs in nucleus accumbens core (NAcC) medium spiny neurons (MSNs). We found synaptic changes after protracted withdrawal from multiple opioids, which were most pronounced in D1-expressing MSNs, and were prevented by CA4 disruption. Moreover, AZD reversed withdrawal-induced synaptic alterations both in vitro and in vivo, in a CA4- and acid-sensing ion channel 1A (ASIC1A)–dependent manner. Unlike withdrawal from cocaine, withdrawal from oxycodone did not alter dendritic spine density in NAcC MSNs, suggesting a distinct mode of plasticity. Finally, following oxycodone self-administration, both CA4 deletion and a single systemic AZD dose reduced drug-seeking after prolonged abstinence. Together, these findings identify CA4 as a regulator of opioid-induced synaptic adaptations and suggest AZD as a promising, readily translatable pharmacological intervention. By targeting a pathway independent of classical opioid receptor signaling, CA4 inhibition represents a mechanistically distinct strategy that may reduce relapse vulnerability in OUD.

## Introduction

The opioid crisis is ongoing, and overdose deaths remain unacceptably high (https://www.cdc.gov/nchs/nvss/vsrr/drug-overdose-data.htm). Opioids and their withdrawal produce a sustained, increasing desire for the drug, referred to as opioid-seeking or craving [1]. Few medications are FDA-approved to treat opioid use disorder (OUD), and all target the mu opioid receptor [2]. While these medications mitigate withdrawal and reduce relapse risk, they fail to correct the persistent synaptic rearrangements that underpin relapse. Thus, new treatments with mechanisms distinct from the mu opioid receptor could have a substantial impact on the clinical management of OUD.

The nucleus accumbens (NAc) is a central hub in the brain circuits underlying responses to drugs of abuse and is thought to play a key role in OUD [3, 4]. ~95% of neurons in NAc are medium spiny neurons (MSNs) [5, 6], which are critical for drug-seeking behaviors [7, 8]. Drugs of abuse, including cocaine and opioids, produce changes in synapses onto NAc MSNs that persist beyond acute drug exposures and may thus bias neural circuits towards future drug-seeking, promoting vulnerability to relapse [9–13]. For example, withdrawal from cocaine has been reported to increase AMPAR/NMDAR ratio at synapses in NAc core (NAcC) [14–18] and shell (NAcS) [11, 14, 16, 19], and to increase Ca^2+^-permeable AMPARs (CP-AMPARs) in NAcC [18, 20–24] and NAcS [11, 14, 24–26]; and such changes have been suggested to promote cocaine-seeking [11, 21, 23, 25–28]. Synaptic responses to opioids are less well-characterized, although similar rearrangements have been reported. For example, withdrawal from non-contingent morphine increased AMPAR/NMDAR ratio in D1^+^ MSNs in NAc shell [29], and withdrawal from non-contingent morphine, heroin, and oxycodone increased AMPAR/NMDAR ratio in NAcC MSNs [30]. Increases in CP-AMPARs have been reported following withdrawal from morphine [29, 31], fentanyl [32], and oxycodone [33, 34]. Supporting an important role for synaptic AMPARs in NAcC in promoting opioid-seeking behaviors, AMPAR antagonists delivered to the NAcC reduced reinstatement in rats following heroin self-administration [9].

We recently identified acid-sensing ion channels (ASICs) as a novel molecular mechanism in cocaine and opioid-seeking behaviors [17, 30]. ASICs are cation channels activated by extracellular acidosis and comprise trimeric assemblies of combinations of ASIC1A, ASIC2A, and ASIC2B subunits [35]. The ASIC1A subunit is required for activation by acidic extracellular pH within a physiologically relevant range (pH 7.2–pH 5) [35]. In NAcC MSNs, ASICs are activated by protons released from glutamate-containing presynaptic vesicles during synaptic transmission [17, 18]. ASIC1A disruption increased sensitivity to synaptic rearrangements induced by cocaine and opioids, and also increased conditioned place preference (CPP) to cocaine and opioids [17, 18, 30]. Together, these findings suggest the possibility that increasing ASIC function might oppose synaptic and behavioral responses to cocaine and opioids. Consistent with this possibility, overexpressing ASIC1A in NAcC in rats reduced cocaine self-administration. Subsequent work, however, suggested that overexpressing ASIC1A in NAcC in rats following cocaine self-administration and withdrawal increased reinstatement of cocaine-seeking [36]. Thus, behavioral effects of potentiating ASIC function may be complex and depend on timing and neuron specificity.

Another strategy for increasing ASIC activation is to reduce pH buffering. Extracellular pH is buffered by the reaction (H^+^ + HCO_3_^−^ ↔ CO_2_ + H_2_O) catalyzed by carbonic anhydrases (CA). Among more than 14 isoforms in mammals [37, 38], we focused on CA4 as a candidate for regulating synaptic ASICs based on its established characteristics. CA4 is abundantly expressed in brain neurons, including NAc MSNs, is anchored in the cell membrane facing the extracellular compartment, and has been previously suggested to buffer synaptic pH [39, 40]. Indeed, disrupting CA4 in postsynaptic MSNs increased acidosis and ASIC-mediated EPSCs [18]. The CA4 inhibitor acetazolamide (AZD) also increased acidosis and potentiated ASIC-mediated EPSCs, and had no added effect on ASIC-mediated EPSCs in *Car4*^−^^*/*^^−^ mice, suggesting actions of AZD on ASICs depend on CA4 [17]. Furthermore, loss of CA4 prevented synaptic rearrangements following withdrawal from cocaine, including increased AMPAR/NMDAR ratio, CP-AMPARs, mEPSC frequency, and dendritic spine density [17, 18]. Consistent with these synaptic effects of CA4 disruption, there were also behavioral effects. *Car4*^*−/−*^ mice self-administered a similar amount of cocaine as *Car4*^*+/+*^ mice, though after 4 weeks of abstinence, *Car4*^*−/−*^ mice had reduced active lever presses (unreinforced by drug) [18]. CA4 disruption also reduced locomotor responses to acute cocaine challenge following withdrawal from cocaine [18]. Here, we investigated the effects of CA4 disruption in mice on opioid withdrawal-induced synaptic adaptations and dendritic spine morphology in NAcC MSNs. We also assessed oxycodone-seeking behavior using an operant model resembling a procedure in rats that produced incubation of oxycodone-seeking behavior [33]. Additionally, we tested pharmacological inhibition of CA4 with acetazolamide (AZD), a carbonic anhydrase inhibitor used clinically for a variety of illnesses [41–43]. We hypothesized that CA4 disruption and pharmacological inhibition would protect against the effects of withdrawal from oxycodone on glutamatergic synapses in NAcC and drug-seeking behavior. Our results suggest AZD may offer a new pharmacological agent for mitigating drug-seeking and relapse in OUD that is unlike traditional opioid replacement therapies that target the mu opioid receptor.

## Materials and methods

### Mice

All mice were on a C57BL/6 genetic background. *Car4*^−/−^ mice (stock no. 008217) [39] and *Drd1a-tdTomato* mice (stock #016204) were obtained from Jackson Laboratory. *Asic1a*^−/−^ mice were generated as described [44]. Mice were housed in groups of 2–5 littermates with free access to standard chow and water. Mice were maintained on a 12-h light-dark cycle; experiments were performed during the light phase. Experimental groups were matched by sex and age (10–15 weeks). Experimentally naïve mice were randomly assigned to conditions. All procedures were approved by the University of Iowa Animal Care and Use Committee and complied with NIH guidelines.

### Oxycodone, heroin, morphine, and AZD

Oxycodone, heroin, and morphine were supplied by NIDA. Acetazolamide (XGen, USA) was purchased from the University of Iowa Hospital pharmacy. Timing, dosing, and delivery of drugs are described in figures.

### Slice preparation and electrophysiology

Following previous protocols [17, 18, 30, 45], 300 µm coronal NAc slices were prepared from mice (8-12 weeks old) in cold buffer (in mM: 225 Sucrose, 26 NaHCO_3_, 1.2 KH_2_PO_4_, 1.9 KCl, 10 D-glucose, 1.1 CaCl_2_, 2 MgSO_4_) with 95% O_2_ and 5% CO_2_. Slices were transferred to oxygenated ACSF (in mM: NaCl 127, 26 NaHCO_3_, 1.2 KH_2_PO_4_, 1.9 KCl, 10 D-glucose, 2.2 CaCl_2_, 2 MgSO_4_) held at 32 °C for 30-min followed by 30-min room temperature. Whole-cell recordings were performed in NAcC MSNs using 2.5–4 MΩ pipettes. Data were obtained with an Axopatch 200B amplifier (Axon Instruments) and analyzed offline by Clampfit (Axon software). Internal solution mM: 125 cesium methanesulfonate, 20 CsCl, 10 NaCl, 2 Mg-ATP, 0.3 Na-GTP, 10 HEPES, 0.2 EGTA, 2.5 QX314, pH 7.3 adjusted with CsOH. EPSCs were evoked using a bipolar tungsten electrode positioned ~200 µm from the neuron. Stimulation intensity was adjusted to ~half-maximum EPSC response. For AMPAR/NMDAR ratios, peak AMPAR-EPSCs were measured at −70 mV and NMDAR-EPSCs were measured 60-ms after onset at +50 mV [46] with CNQX (20 µM) added to the bath. Picrotoxin (100 µM) was added for all recordings. Alternative assessment of AMPAR/NMDAR ratios using peak NMDAR currents preserved effects of CA4 and AZD (Fig. S1). AMPAR rectification was determined by recording at −70, −30, +30, and +50 mV in the presence of picrotoxin (100 µM), APV (100 µM). AMPAR rectification index was calculated as the ratio of current at −70 mV to +50 mV. For NASPM sensitivity, cells were held at −70 mV, with 20–25 baseline sweeps collected in the presence of picrotoxin (100 µM) and APV (100 µM). NASPM (200 μM, Alomone Lab) was applied, and 15-min later, 20–25 sweeps were collected. In vitro effects of CA4 inhibition were tested by applying AZD (100 μM) for 1-h.

### DiI labeling, dendritic spine imaging, and analysis

Mice were perfused, coronal slices cut, and NAcC MSNs stained with DiI, as previously described [18, 45]. Dendritic segments were imaged, and spine density and morphology were analyzed using Neuron Studio, as previously described [18, 45]. Each experimental group consisted of 3–4 animals, with a total of 9–16 neurons/group, 3–4 neurons analyzed/animal. Each neuron was averaged over 2–4 dendritic segments, with each segment ~50–60 µm in length. Results were calculated as average density (number of spines/µm dendritic length) per neuron. Number of spines assessed by group: *Car4*^+/+^ saline-veh (3850), *Car4*^+/+^ saline-AZD (4646), *Car4*^+/+^ oxycodone-veh (3007), and *Car4*^+/+^ oxycodone-AZD (3960).

### Oxycodone conditioned place preference (CPP)

Oxycodone CPP was as previously described [30]. In brief, mice underwent pre- and post-tests when they were allowed to explore a two-chamber CPP apparatus (Med Associates) for 20-min. For 3 days between pre- and post-tests, mice underwent two training sessions daily, during which oxycodone (15 mg/kg, i.p.) and saline were paired with opposite compartments. Preference on the oxycodone-paired side was calculated by subtracting the pre-test from the post-test.

### Oxycodone self-administration (SA)

First, mice underwent jugular vein catheterization [18]. Catheter patency was verified post-surgery and during the course of the SA protocol. Mice were fasted overnight prior to SA training and restricted to 85–90% pre-fasting bodyweight during days 1–10 [18]. Training occurred in operant chambers (Med Associates) equipped with cues (light and tone) and two levers (active vs. inactive). Mice received oxycodone (0.25 mg/kg/infusion) on a fixed-ratio 1 (FR1) schedule during daily 6-h sessions for 10 days minimum, as described [47]. Active lever presses (ALPs) triggered oxycodone infusions and 20 s of cues during which no additional infusions could be obtained. Mice meeting the criteria of at least 10 infusions/day for the final 3 days were included in further testing. 24-h after the final SA session, baseline drug-seeking was assessed by returning mice to operant chambers for 30-min during which ALPs triggered cues but no drug. After 30 days of forced abstinence, mice received AZD (30 mg/kg, i.p.) or vehicle (saline). 24-h later, drug-seeking behavior was again assessed as at baseline.

### Statistical analyses

Student’s t-test was used to assess differences between two groups, while two-way ANOVA was used to assess significance for experiments involving 2 independent factors. Within the context of the ANOVA, planned contrast testing (Fisher’s LSD) was used to test a priori hypothesized relationships between groups. To assess the significance of lever press data following oxycodone SA, three-way ANOVA with repeated measures was used to compare between-subject factors (genotype, AZD) and within-subject factors (time). Paired-sample t-tests were used to compare the same groups of mice tested at two different time points (baseline vs post-withdrawal). ROUT (Q = 1%) was used to screen for outliers. The F-test was used to compare variances between groups, and Welch’s correction was applied for unequal variances. P < 0.05 was considered significant. Bar graphs express values as mean ± S.E.M. Statistical analyses were performed using GraphPad Prism. Detailed statistical data are given in Tables S1 and S2.

## Results

### CA4 disruption prevented oxycodone-withdrawal-induced increases in AMPAR/NMDAR ratio and CP-AMPARs, and attenuated CPP

**Fig. 1:**
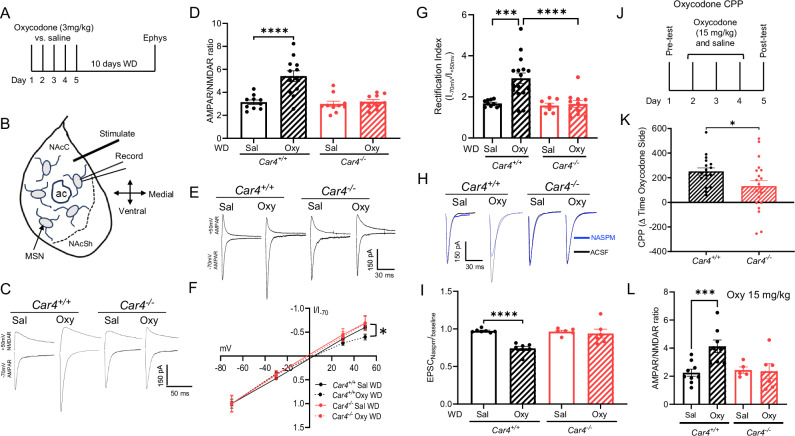
CA4 disruption protects against oxycodone withdrawal-induced synaptic changes at glutamatergic synapses in NAcC MSNs and reduces oxycodone conditioned place preference (CPP). **A** Experimental timeline: oxycodone (3 mg/kg, i.p.) or saline (i.p.) was administered in the home cage each day for 5 days, followed by 10 days of withdrawal, after which slice electrophysiology was performed. **B** Diagram illustrating the location of MSN recordings in NAcC and electrical stimulation. ac anterior commissure, NAcSh NAc shell. **C** Representative traces of the AMPAR-mediated EPSC at −70 mV and the NMDAR-mediated EPSC at +50 mV from NAcC MSNs from *Car4*^*+/+*^ and *Car4*^−^^*/*^^−^ mice following withdrawal from oxycodone (Oxy) vs saline (Sal). **D** AMPAR/NMDAR ratio increased after withdrawal from oxycodone (Oxy) in *Car4*^*+/+*^ but not in *Car4*^−^^*/*^^−^ (n = 10–12 neurons from 5 mice/group). **E** Example traces of AMPAR-mediated EPSCs at -70 mV and +50 mV in NAcC MSNs of *Car4*^*+/+*^ and *Car4*^−^^*/*^^−^ mice following withdrawal from oxycodone (Oxy) vs saline withdrawal (Sal). **F** Current-voltage relationship (IV-curve): withdrawal from oxycodone produced inward rectification in *Car4*^*+/+*^ mice (n = 7–16 neurons from 3 to 5 mice/group). **G** Quantification of rectification index shows an increase after withdrawal from oxycodone in *Car4*^*+/+*^ but not in *Car4*^−^^*/*^^−^ mice (n = 7–16 neurons from 3 to 5 mice/group). **H** Representative traces of the AMPAR-mediated evoked EPSC at −70 mV before (black) and after (blue) NASPM treatment. **I** NASPM sensitivity increased after withdrawal from oxycodone in *Car4*^*+/+*^ but not in *Car4*^−^^*/*^^−^ mice (n = 6–7 neurons from 3 mice/group). **J** Experimental timeline for oxycodone CPP in *Car4*^*+/+*^ and *Car4*^−^^*/*^^−^ mice. **K** Oxycodone conditioned place preference is impaired in *Car4*^−^^*/*^^−^ mice (n = 18, 20 mice/grp). **L** *Car4*^*+/+*^ and *Car4*^−^^*/*^^−^ mice were administered oxycodone (15 mg/kg, i.p.) or saline (i.p.) in the home cage each day for 5 days, followed by 10 days of withdrawal, after which slice electrophysiology was performed. AMPAR/NMDAR ratio increased after withdrawal from oxycodone (Oxy) in *Car4*^*+/+*^ but not in *Car4*^−^^*/*^^−^ (n = 5–9 neurons from 2 to 3 mice/group).

To test whether oxycodone withdrawal induces synaptic rearrangements that are sensitive to CA4 disruption, we delivered oxycodone (3 mg/kg, i.p.) vs. saline control once daily for 5 days to *Car4*^+/+^ and *Car4*^–/–^ mice and then withheld oxycodone for 10 days (Fig. 1A). We then harvested brain tissue and tested glutamatergic transmission onto NAcC MSNs in acute slices (Fig. 1B). We found that withdrawal from oxycodone increased AMPAR/NMDAR ratio in *Car4*^*+/+*^ mice but not in *Car4*^–/–^ mice (Fig. 1C, D; drug × genotype interaction, F(1,38) = 10.55, p = 0.0024), suggesting loss of CA4 prevented the oxycodone-induced change. Effects of oxycodone and CA4 disruption were independent of sex (Fig. S2). To test if extended withdrawal was required to increase the AMPAR/NMDAR ratio, we tested 24 h of withdrawal after 5 doses (once daily × 5 days) and after one dose (Fig. S3A). These 24-h withdrawal periods were insufficient to change AMPAR/NMDAR ratios (Fig. S3B), suggesting an extended withdrawal period was required.

An increase in GluA2-lacking CP-AMPARs in the postsynaptic membrane is one mechanism that may contribute to increased AMPAR/NMDAR ratios [11, 14, 18]. CP-AMPARs are more inwardly rectifying [48, 49]; therefore, we tested rectification index in NAcC MSNs by measuring current-voltage relationships of synaptic AMPAR responses (Fig. 1E). Indeed, withdrawal from oxycodone increased rectification index in *Car4*^+/+^ mice (vs. saline) but not in *Car4*^−/−^ mice (Fig. 1E, F, G; drug x genotype interaction, F(1,40) = 6.625, p = 0.0139). We also tested sensitivity to the CP-AMPAR-specific antagonist NASPM and found increased NASPM sensitivity in *Car4*^+/+^ mice following withdrawal from oxycodone (Fig. 1H, I; drug x genotype interaction, F(1,22) = 10.40, p = 0.0039). Consistent with a protective effect of CA4 disruption, NASPM sensitivity was unchanged in *Car4*^−/−^ mice. Together, these findings suggest that withdrawal from oxycodone increases CP-AMPARs in the postsynaptic membrane of NAcC MSNs and that loss of CA4 prevents this increase. These findings highlight CA4 disruption as a potential strategy for preventing synaptic rearrangements associated with withdrawal from oxycodone.

To assess whether CA4 disruption affects drug-associated memory, we tested oxycodone CPP (15 mg/kg, i.p.) (Fig. 1J), a dose that produced exaggerated CPP in *Asic1a*^*–/–*^ mice [30]. We found that both *Car4*^+/+^ and *Car4*^*–/–*^ mice preferred the oxycodone-paired chamber, although this preference was reduced in *Car4*^*–/–*^ mice (Fig. 1K, t(31.31) = 2.197, p = 0.0355). We further tested the effects of this oxycodone dose on the AMPAR/NMDAR ratio; we administered oxycodone (15 mg/kg) vs saline daily for 5 days, followed by 10 days of withdrawal. We found an increase in AMPAR/NMDAR ratio in oxycodone-withdrawn *Car4*^*+/+*^ mice vs. saline-treated controls, which was absent in *Car4*^−^^*/*^^−^ mice (Fig. 1L; drug × genotype interaction, F(1,24) = 5.987, p = 0.0221). Together, these results indicate CA4 disruption attenuates oxycodone CPP and protects against synaptic rearrangements following withdrawal from oxycodone.

### AZD reversed CA4-dependent oxycodone-induced synaptic changes

To explore whether pharmacologically inhibiting CA4 produces effects similar to CA4 disruption, we used AZD [40, 50]. AZD is approved to treat a variety of illnesses in humans [41–43, 51], and its properties make it ideal. AZD potently blocks CA4 (~10 nM affinity), as well as other CAs [52, 53]. AZD readily crosses the blood–brain barrier [54]. It is rapidly metabolized and excreted by the kidneys, with a ~1 h half-life in mice [55, 56]. Moreover, AZD potentiates ASIC1A-mediated synaptic currents in NAcC MSNs in a CA4-dependent manner [17, 18].

To determine whether AZD produces effects similar to CA4 disruption, we first tested its effects in brain slices in *Car4*^*+/+*^ mice. We delivered oxycodone (3 mg/kg, i.p. vs. saline) daily for 5 days, followed by 5 days of withdrawal (Fig. S4A). We then assessed the AMPAR/NMDAR ratio in NAcC MSNs. As before, withdrawal from oxycodone increased the AMPAR/NMDAR ratio. Compared to the vehicle, applying AZD (100 μM) to the bath for 1 h reduced the AMPAR/NMDAR ratio in oxycodone-withdrawn mice (Fig. S4B, oxy × AZD interaction, F(1,37) = 8.729, p = 0.0054). Importantly, AZD had no effect on the AMPAR/NMDAR ratio in saline-treated mice. These data suggest that acute AZD application normalizes opioid-induced increases in AMPAR/NMDAR ratio within 1 h.

We next tested whether administering AZD in vivo has similar effects. We estimated an AZD dose of 30 mg/kg, i.p., should achieve a systemic concentration similar to that used in our brain slice experiments and analogous to that used in humans [57]. We administered oxycodone (3 mg/kg, i.p. vs. saline) daily for 5 days. After 5 days of withdrawal (Fig. S4C), we gave AZD vs. vehicle. Three hours later, we harvested brain slices and assessed the AMPAR/NMDAR ratio in NAcC MSNs. Similar to our observations in vitro, administering AZD in vivo after withdrawal from oxycodone reduced AMPAR/NMDAR to levels comparable to those of vehicle-treated saline-withdrawn counterparts **(**Fig. S4D, oxycodone x AZD interaction, F(1,26) = 21.69, p < 0.0001**)**, while AZD had no effect in mice not exposed to oxycodone. To test generalizability to other opioids, we also tested effects of in vivo AZD administration after 5 days of withdrawal from morphine and observed similar results (Fig. S4F, Mor × AZD interaction, F(1,24) = 10.34, p = 0.0196).

**Fig. 2:**
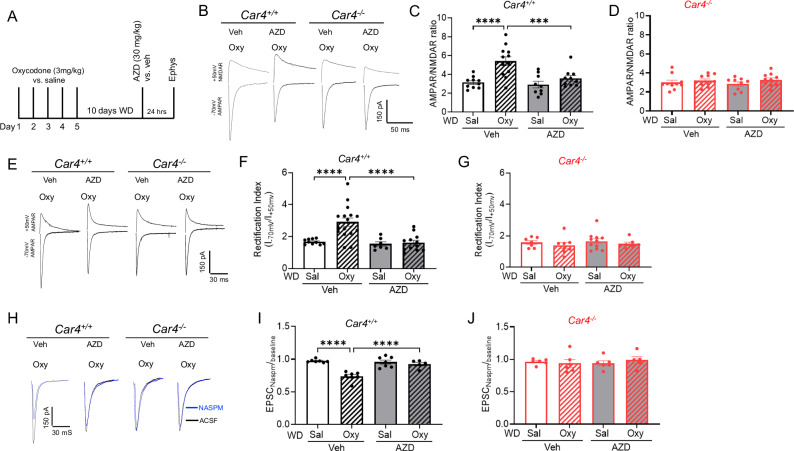
Acetazolamide (AZD) reversed oxycodone withdrawal-induced changes at glutamatergic synapses in NAcC MSNs in *Car4*^*+/+*^ mice but not in *Car4*^*–/–*^ mice. **A** Experimental timeline: oxycodone (3 mg/kg, i.p.) or saline (i.p.) was administered each day for 5 days, followed by 10 days of withdrawal. AZD (30/mg/kg, i.p.) or vehicle (saline, i.p.) was administered, and 24-h later, slices were harvested for electrophysiological recording. **B** Representative traces of AMPAR/NMDAR from *Car4*^*+/+*^ and *Car4*^−^^*/*^^−^ mice after withdrawal from oxycodone (Oxy) and treatment with AZD vs. vehicle (Veh). **C** AZD significantly reduced oxycodone withdrawal-induced increase in AMPAR/NMDAR ratio in *Car4*^*+/+*^ mice (n = 9–12 neurons from 3 to 4 mice/group). **D** Withdrawal from oxycodone and AZD did not affect AMPAR/NMDAR ratio in *Car4*^−^^*/*^^−^ mice (n = 9–10 neurons from 3 to 4 mice/group). **E** Representative traces of AMPAR-mediated EPSCs at −70 and +50 mV from oxycodone-withdrawn *Car4*^*+/+*^ and *Car4*^−^^*/*^^−^ mice after treatment with AZD vs. vehicle. **F** AZD treatment attenuated oxycodone withdrawal-induced increase in AMPAR rectification in *Car4*^*+/+*^ mice (n = 7–16 neurons from 3 to 5 mice/group). **G** Oxycodone withdrawal and AZD had no effect on AMPAR rectification in *Car4*^−^^*/*^^−^ mice (n = 7–11 neurons from 3 to 4 mice/group). **H** Representative traces of the AMPAR-mediated evoked EPSC at −70 mV before (black) and after (blue) NASPM application from *Car4*^*+/+*^ and *Car4*^−^^*/*^^−^ mice after withdrawal from oxycodone (Oxy) and treatment with AZD vs. vehicle. **I** AZD treatment attenuated oxycodone withdrawal-induced increase in NASPM sensitivity in *Car4*^*+/+*^ (n = 6–7 neurons from 3 mice/group). **J** withdrawal from oxycodone and AZD had no effect on NASPM sensitivity in *Car4*^−^^*/*^^−^ mice (n = 5–6 neurons from 3 mice/group).

Next, we tested whether the effects of in vivo-administered AZD lasted beyond 3 h. We administered AZD vs. vehicle following withdrawal from oxycodone and tested AMPAR/NMDAR 24 h later (Fig. 2A). Withdrawal from Oxycodone again increased the AMPAR/NMDAR ratio in *Car4*^+/+^ mice, and importantly, AZD reduced it back to baseline levels (Fig. 2B, C; oxycodone x AZD interaction, F(1,37) = 4.966, p = 0.032). As with CA4 disruption, AZD effects were sex-independent (Fig. S2). Together, these findings suggest that AZD reverses opioid-induced synaptic rearrangements. Moreover, because of its short half-life, the effects of AZD likely persisted beyond its clearance [55, 56].

We hypothesized that AZD effects on AMPAR/NMDAR ratio were mediated through CA4 inhibition rather than another target. Therefore, we also tested AZD in *Car4*^*–/–*^ mice and found no effect of oxycodone or AZD on AMPAR/NMDAR ratio (Fig. 2D**)**. We similarly tested effects of AZD on oxycodone-withdrawal-induced changes in AMPAR rectification and NASPM sensitivity. In *Car4*^*+/+*^ mice, AZD reversed oxycodone-associated increases in both rectification (Fig. 2E, F; oxy × AZD interaction, F(1,41) = 6.862, p = 0.0123) and NASPM sensitivity (Fig. 2H, I, oxy × AZD interaction, F(1,23) = 18.38, p = 0.0003), suggesting that AZD also reverses oxycodone-induced increases in CP-AMPARs. In contrast, in *Car4*^−/−^ mice, neither rectification index or NASPM sensitivity was affected by withdrawal from oxycodone or AZD (Fig. 2E, G, H, J). These results are consistent with our hypothesis; however, because there were no oxycodone-induced changes to reverse in *Car4*^−/−^ mice, the absence of an AZD effect in this background cannot alone establish causality, but it supports the interpretation that AZD acts through CA4 rather than an off-target mechanism.

### AZD also reversed changes in AMPAR/NMDAR induced by heroin and morphine

**Fig. 3:**
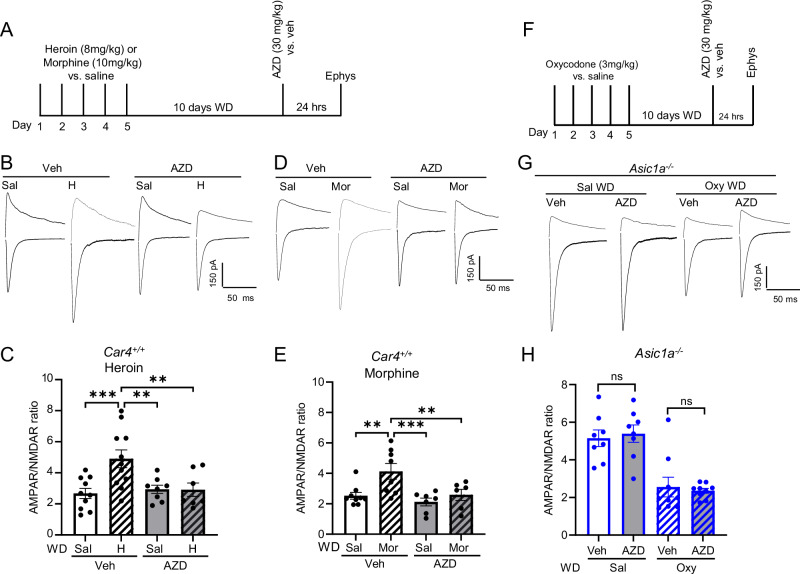
Effects of AZD on oxycodone-induced increases in AMPAR/NMDAR depend on ASIC1A and generalize to other opioids. **A** Experimental timeline for heroin, morphine, and AZD treatment. **B** Representative traces of AMPAR/NMDAR from *Car4*^*+/+*^ mice after heroin (H) or saline (Sal) withdrawal and treatment with AZD vs vehicle. **C** AZD treatment reversed the heroin-induced increase in AMPAR/NMDAR ratio relative to vehicle-treated controls (n = 8–11 neurons from 4 mice/group). **D** Representative traces of AMPAR/NMDAR from *Car4*^*+/+*^ mice after withdrawal from morphine (Mor) or Saline (Sal) and treatment with AZD vs. vehicle. **E** AZD treatment reversed morphine-induced increase in AMPAR/NMDAR ratio compared to vehicle-treated controls (n = 7–9 neurons from 4 mice/group). **F** Experimental timeline: oxycodone (3 mg/kg, i.p.) or saline (i.p.) was administered each day for 5 days, followed by 10 days of withdrawal. AZD or vehicle was administered, and 24-h later, slices were harvested for electrophysiological recording. **G** Representative traces of AMPAR/NMDAR from *Asic1a*^−^^*/*^^−^ mice withdrawn from oxycodone (Oxy) or saline (Sal) and treated with AZD vs. vehicle. **H** AZD had no effect in drug naïve and oxycodone-withdrawn *Asic1a*^−^^*/*^^−^ mice (n = 8–10 neurons from 4 mice/group).

To test whether the protective effects of AZD generalized to other opioids, we also tested withdrawal from heroin (8 mg/kg for 5 days) and morphine (10 mg/kg for 5 days) (Fig. 3A). After 10 days of withdrawal, *Car4*^*+/+*^ mice received AZD or vehicle, and 24 h later AMPAR/NMDAR ratio was assessed. Withdrawal from heroin (Fig. 3B, C) and morphine (Fig. 3D, E) both increased AMPAR/NMDAR ratio in NAcC MSNs (heroin, F(1,32) = 6.156, p = 0.0006; morphine, F(1,26) = 7.179, p = 0.0025), and AZD reversed these effects.

Withdrawal from a lower heroin dose (2 mg/kg, i.p.) did not increase AMPAR/NMDAR ratio (Fig. S5A, B), suggesting that dose matters for at least some of the opioid-induced synaptic rearrangements. In another experiment, we tested just 5 days of withdrawal from morphine (10 mg/kg) and found it was sufficient to increase AMPAR/NMDAR (Fig. S6A, B) (F(1,36) = 2.697, p = 0.0102), suggesting that withdrawal periods as short as 5 days, but longer than 24 h, are sufficient to alter the AMPAR/NMDAR ratio. Together, these observations suggest that synaptic adaptations evoked by withdrawal from oxycodone and the normalizing effects of AZD are not specific to oxycodone but also generalize to other opioids.

### AZD had no effect on AMPAR/NMDAR ratios in *Asic1a*^−^^*/*^^−^ mice

We next tested withdrawal from oxycodone and AZD in *Asic1a*^−^^*/*^^−^ mice. We previously reported that *Asic1a*^−^^*/*^^−^ mice have an elevated AMPAR/NMDAR at baseline, and exposure to cocaine or oxycodone normalizes it towards baseline levels seen in drug-naive *Asic1a*^*+/+*^ controls [17, 18, 30, 45]. Thus, we wondered whether AZD could impact either the elevated baseline AMPAR/NMDAR ratio in *Asic1a*^−^^*/*^^−^ mice or the oxycodone-induced reduction. We delivered oxycodone (3 mg/kg, i.p. vs. saline) daily for 5 days, followed by 10 days of withdrawal, then treated with AZD vs. vehicle, and 24 h later tested the AMPAR/NMDAR ratio in NAcC MSNs. AZD had no effect on either the elevated baseline or the oxycodone-withdrawn AMPAR/NMDAR ratios (Fig. 3G, H). Together, these results suggest AZD’s ability to reduce AMPAR/NMDAR ratio depends on ASIC1A and are consistent with the actions of AZD and CA4 on ASIC1A-mediated currents [17].

### Effects of oxycodone withdrawal and AZD on AMPAR/NMDAR were observed in D1^+^ MSNs but not D1^–^ MSNs

**Fig. 4:**
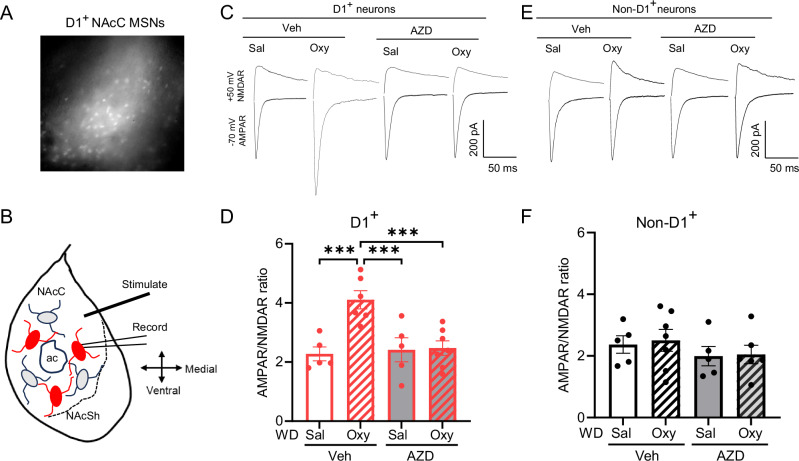
Increase in AMPAR/NMDAR ratio in oxycodone-withdrawn mice was specific to D1^+^ neurons. **A** Image of D1^+^ neurons in NAcC labeled with tdTomato. **B** Diagram illustrating the location of recordings of D1^+^ and non-D1^+^ MSNs in NAcC. **C** Representative traces of AMPAR/NMDAR ratio of D1^+^ neurons in oxycodone-withdrawn (Oxy) and saline-withdrawn (Sal) mice treated with AZD vs. vehicle. **D** Oxycodone-withdrawn mice exhibit increases in AMPAR/NMDAR ratio D1^+^ neurons, and AZD administration reversed the measure to levels in vehicle-treated controls (n = 5–7 neurons from 3 mice/group). **E** Representative traces of AMPAR/NMDAR ratio of non-D1^+^ MSNs in oxycodone-withdrawn (Oxy) and saline-withdrawn (Sal) mice treated with AZD vs. vehicle. **F** Oxycodone-withdrawn mice exhibited no change in AMPAR/NMDAR ratio in D1^–^ neurons, and AZD had no effect (n = 5–7 neurons from 3 mice/group).

NAcC MSNs are subclassified by expression of dopamine receptor subtypes (D1 vs. D2) [5, 6]. Conventionally, these MSN subtypes have been thought to play differing roles, with D1^+^ NAcC MSNs promoting drug-seeking behaviors [7, 15, 57, 58], and D2^+^ MSNs opposing drug-seeking [7, 8], although more recent observations suggest more nuanced roles of these neuron types in reward and aversion [59, 60]. To test whether the effects of oxycodone and AZD described above were specific to MSN subtype, we leveraged mice expressing tdTomato selectively in D1^+^ MSNs (Fig. 4A). We tested responses in AMPAR/NMDAR ratio in D1^+^-NAcC MSNs versus non-D1^+^-NAcC MSNs (not expressing tdTomato) using the same oxycodone and AZD exposures as before (Fig. 2A). We recorded from D1^+^- and non-D1^+^- NAcC MSNs as illustrated (Fig. 4B). We found withdrawal from oxycodone evoked an increase in AMPAR/NMDAR ratio in D1^+^ MSNs which was reversed by AZD (Fig. 4C, D, oxy x AZD interaction, F(1,19) = 8.578, p = 0.0086). However, in non-D1^+^ NAcC MSNs, withdrawal from oxycodone did not change the AMPAR/NMDAR ratio, and AZD had no additional effect (Fig. 4E, F). Potential effects on CP-AMPARs were not tested. These results suggest effects of withdrawal from oxycodone on AMPAR/NMDAR ratios are more selective for D1^+^-MSNs.

### Dendritic spine density in NAcC MSNs was unaffected by withdrawal from oxycodone and AZD

Along with functional changes in glutamatergic synapses in the NAcC (e.g., AMPAR/NMDAR ratio), changes in dendritic spine density following withdrawal from cocaine have also been reported. However, the direction and magnitude of spine changes have varied across studies and exposure procedures, and causal relationships between synaptic physiology, spine densities, and drug-seeking behaviors have not been clearly established [61–64]. Fewer studies have investigated the effects of withdrawal from opioids on dendritic spine density, leaving open the question of whether withdrawal from opioids produces structural changes similar to those reported with cocaine. Supporting differing effects of opioids versus cocaine, we recently observed no effects of withdrawal from oxycodone on spine density in either wild-type or mice lacking ASIC1A [30, 63]. Thus, we were interested in whether inhibiting CA4 with AZD would affect dendritic spine density following withdrawal from opioids. To test this possibility, we administered oxycodone for 5 days, followed by 10 days of withdrawal. We then administered AZD or vehicle and 24 h later harvested brain tissue for dendritic spine analyses (Fig. S7A). We found that total spine density was similar between oxycodone- and saline-withdrawn *Car4*^*+/+*^ mice (Fig. S7B, C), with no differences in the density of stubby (Fig. S7D), thin (Fig. S7E), or mushroom spines (Fig. S7F). These results were consistent with our recently reported absence of effect of withdrawal from oxycodone on dendritic spine density in NAcC MSNs [30]. Importantly, in both oxycodone- and saline-withdrawn mice, AZD had no effect on these measures versus vehicle (Fig. S7B–F). Together, these results suggest that withdrawal from oxycodone exerted little or no effect on dendritic spine density in NAcC MSNs, and thus differ from effects we have recently seen following a similar protocol of withdrawal from cocaine [18]. In addition, these data suggest that the above alterations in glutamatergic neurotransmission evoked in NAcC MSNs by withdrawal from oxycodone were not accompanied by changes in dendritic spine density.

### Drug-seeking and elevated AMPAR/NMDAR ratios following withdrawal from oxycodone SA were reduced by CA4 disruption and AZD

**Fig. 5:**
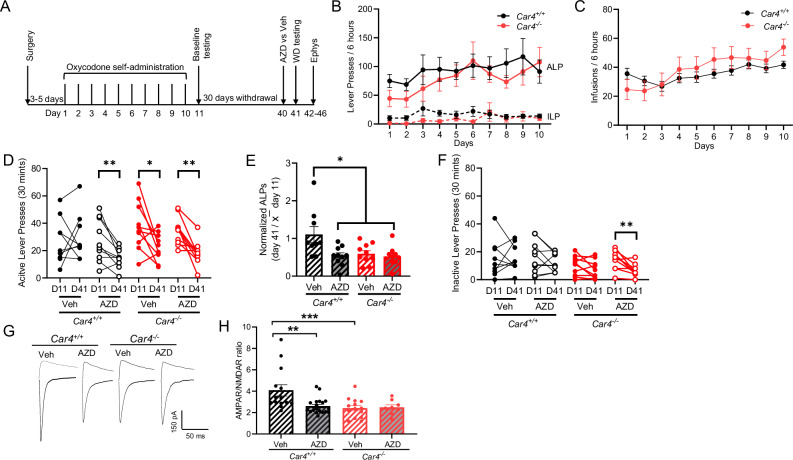
CA4 disruption and inhibition reduced cue-induced drug-seeking behavior following forced abstinence from oxycodone self-administration. **A** Experimental timeline for intravenous oxycodone self-administration and slice electrophysiology. **B** Lever presses (active (ALP) and inactive (ILP) across experimental sessions. There was no effect of genotype (n = 10 mice/per group). **C** Infusions across experimental sessions between the genotypes. There was no effect of genotype. **D** Active lever presses at baseline (day 11) and after 30 days of forced abstinence (day 41). There was no significant time by AZD × genotype interaction, though there was a significant time × genotype interaction. In vehicle-treated *Car4*^*+/+*^ mice, active lever presses did not change from day 11 to day 41 (n = 9). In AZD-treated *Car4*^*+/+*^ mice (n = 10), vehicle-treated *Car4*^−^^*/*^^−^ mice (n = 10), and AZD-treated *Car4*^−^^*/*^^−^ mice (n = 10), lever presses decreased from day 11 to day 41. **E** Day 41 lever presses normalized to mean day 11 lever pressing. Two-way ANOVA identified a significant genotype × AZD interaction. Planned contrast testing revealed that vehicle-treated *Car4*^*+/+*^ mice had greater cue-induced oxycodone seeking than AZD-treated *Car4*^*+/+*^ mice, vehicle-treated *Car4*^−^^*/*^^−^ mice, and AZD-treated *Car4*^−^^*/*^^−^ mice. **F** Inactive lever presses at baseline (day 11) and after 30 days of forced abstinence (day 41). There was no significant time by AZD × genotype interaction. Inactive lever pressing did not change significantly between day 11 and day 41 in vehicle-treated *Car4*^*+/+*^ mice, AZD-treated *Car4*^*+/+*^ mice, and vehicle-treated *Car4*^−^^*/*^^−^ mice. Inactive lever pressing decreased in AZD-treated *Car4*^−^^*/*^^−^ mice. **G** Representative traces of AMPAR (−70 mV) and NMDAR (+50 mV) from NAcC of *Car4*^*+/+*^ and *Car4*^−^^*/*^^−^ mice treated with AZD vs. vehicle and recorded after forced abstinence from oxycodone self-administration and behavioral testing of drug-seeking. **H** AZD treatment reversed the AMPAR/NMDAR ratio in *Car4*^*+/+*^ mice without affecting *Car4*^−^^*/*^^−^ mice (n = 8–10 neurons from 3 to 6 mice/group).

To examine potential behavioral consequences of the above-described effects of CA4 disruption and AZD, we next turned to oxycodone SA. SA procedures allow rodents to control the amount of drug they consume, and thus model human drug use and seeking [1, 65]. *Car4*^*+/+*^ mice and *Car4*^−^^*/*^^−^ mice were implanted with jugular catheters for oxycodone SA (0.25 mg/kg/infusion) in response to active lever presses (ALP) on a FR1 schedule. Mice were allowed to SA oxycodone for 6 h daily for 10 days (Fig. 5A). A 30-min baseline test of cue-induced drug-seeking behavior was assessed on day 11, in which ALPs produced the light and tone cues but no drug infusion. After 30 days of abstinence (experimental day 40), mice received a single administration of AZD (30 mg/kg, i.p.) or vehicle and 24-h later (day 41) again underwent a cue-reinforced drug-seeking session for comparison to baseline testing. Overall, we found mice of both genotypes similarly acquired the SA task (days 1 through 10) and both genotypes developed a similar preference for ALPs versus inactive lever presses (ILPs) (Fig. 5B). Additionally, both genotypes received a similar number of oxycodone infusions (Fig. 5C). These results suggest that CA4 disruption did not affect oxycodone consumption. After 30 days of abstinence, vehicle-injected *Car4*^*+/+*^ mice maintained a similar amount of cue-reinforced ALPs relative to the baseline (Fig. 5D), suggesting that the desire to obtain oxycodone was sustained. In contrast, we found effects of CA4 disruption and AZD on ALPs (Fig. 5D**;** genotype × time interaction, F(1,35) = 4.733, p = 0.0364) (Fig. 5E; genotype vs. AZD interaction, F(1,35) = 4.252, p = 0.0467). AZD-injected *Car4*^*+/+*^ mice reduced ALPs by half (Fig. 5D, E, t(9) = 3.333, p = 0.0088). ALPs in *Car4*^−^^*/*^^−^mice were also reduced by half on day 41 relative to baseline testing (day 11) (Fig. 5D, E, t(9) = 2.529, p = 0.0323), and AZD had no additional effects (Fig. 5D, E). ILPs were largely unaffected by abstinence, CA4 disruption, or AZD (Fig. 5F; no interaction effects), although there was a reduction in ILPs following abstinence in *Car4*^*–/–*^ mice treated with AZD (Fig. 5F t(9) = 3.591, p = 0.0058). Together, these data suggest that after 30 days of abstinence, AZD and CA4 disruption both suppressed oxycodone-seeking behavior, and that the effects of AZD depended on CA4.

We next harvested brain tissue following SA on days 42–46 to test whether behavioral effects of AZD and CA4 might be related to oxycodone withdrawal-induced differences in glutamatergic transmission. Indeed, we found that AZD treatment in *Car4*^*+/+*^ mice significantly attenuated the AMPAR/NMDAR ratio in NAcC MSNs compared to non-AZD-treated *Car4*^*+/+*^ mice (Fig. 5G, H, genotype x AZD interaction, F(1,51) = 5.126, p = 0.0278). Importantly, AZD did not impact the AMPAR/NMDAR ratio in *Car4*^−^^*/*^^−^ mice, and the AMPAR/NMDAR ratio was lower in vehicle-treated *Car4*^−^^*/*^^−^ mice compared to vehicle-treated *Car4*^*+/+*^ mice (F(1,51) = 5.126, p = 0.0006). AMPAR rectification and NASPM sensitivity were not tested in this procedure. Together, these results parallel behavioral changes observed during drug-seeking (Fig. 5D, E) and support the possibility that behavioral effects of AZD and CA4 disruption are related to their effects on glutamatergic synapses in NAcC MSNs.

## Discussion

These results provide new insights into synaptic adaptations in NAcC MSNs induced by withdrawal and abstinence from opioids, which may contribute to persistent drug-seeking. These results further reveal a novel role for CA4 in opioid-induced synaptic adaptations and suggest that medications that inhibit CA4, such as AZD, may hold promise for reducing the risk of relapse in people with OUD.

### Increases in CP-AMPARs likely contribute to oxycodone-induced increases in AMPAR/NMDAR ratio but other mechanisms are possible

We found that oxycodone withdrawal increased AMPAR/NMDAR ratio and increased CP-AMPARs at glutamatergic synapses in NAcC MSNs in *Car4*^*+/+*^ mice, assessed via rectification index and NASPM sensitivity. Additionally, CP-AMPARs have a higher conductance [49]. Thus, it is reasonable to conclude that increases in CP-AMPARs likely underlie the increase in AMPAR/NMDAR ratio, at least in part. However, it is also possible that other factors contribute. For example, non-CP-AMPARs or NMDAR-mediated currents could change following drug withdrawal. Additionally, formation of silent synapses or their unsilencing could contribute [66]. Our method for assessing AMPAR/NMDAR provides a reliable comparison between these two EPSC components but does not provide a rigorous quantification of their absolute magnitudes. Miniature EPSCs, quantification of evoked AMPAR- and NMDAR-mediated EPSC components in response to varying stimulus intensities, and assessment of silent synapses would provide important insights into the relative contributions of AMPARs vs. NMDARs to the changes observed here. We previously found increases in AMPAR miniature EPSCs in NAcC MSNs following withdrawal from cocaine that were prevented by CA4 disruption [18], thus bolstering the interpretation that changes in AMPARs contribute to opioid effects observed here.

### Temporal limits, drug-administration contingency, and neural specificity of oxycodone-induced changes

We do not yet know the temporal limits of the withdrawal from oxycodone-induced neuroadaptations, but changes in AMPAR/NMDAR required more than 24-h, emerged within 5 days of withdrawal, and persisted for more than 30 days following oxycodone SA and were thus relatively long-lasting. Previous studies in mice found similar changes in AMPAR/NMDAR ratio and/or CP-AMPARs at 7–10 days post-withdrawal from experimenter-administered cocaine [11, 18, 19]. The above-described effects of oxycodone on AMPAR/NMDAR ratio occurred with both experimenter- and SA drug, suggesting that the effect depended on the drug itself rather than how it was administered. Although we did not assess CP-AMPARs after SA, we might expect a similar increase; recent studies in rats suggest that oxycodone SA increases CP-AMPARs in NAcC MSNs after 15 days of abstinence [33, 34]. Interestingly, analogous studies with psychostimulants in rats suggest increases in CP-AMPARs following SA may follow a slower time course [20, 67]. However, it is also important to consider that differences between our findings of drug-induced effects in mice versus previous studies in rats might also reflect species-dependent differences, drug doses, or withdrawal times.

Here, the increase in AMPAR/NMDAR ratio following withdrawal from oxycodone mapped to D1^+^ MSNs and not to non-D1^+^ MSNs, consistent with earlier views that D1^+^ MSNs promote reward- and drug-seeking behaviors [7, 15, 57, 58]. However, growing evidence suggests that the roles of D1^+^ versus D2^+^ MSNs are not strictly dichotomous. Rather, both populations can contribute to reward- and aversion-related behaviors in a circuit- and context-dependent manner and may function cooperatively within distributed motivational networks [59, 60]. Interestingly, oxycodone incubation in rats was recently reported to increase CP-AMPARs in both D1^+^ and D2^+^ MSNs [33], whereas psychostimulant-induced increases in CP-AMPARs appear more selective to D1^+^ MSNs [11, 23]. Additional work will be required to determine the mechanisms that generate the D1-selective effects observed here and to clarify their implications for drug-seeking behavior.

Our findings that oxycodone effects generalized to other mu receptor agonists, morphine and heroin, are not surprising. More surprising is that our results with these opioids also paralleled, at least in part, those observed previously in NAcC MSNs following abstinence from cocaine [17, 18]. Despite binding to different targets, opioids and cocaine share some converging molecular effects. For example, exposure to both opioids and cocaine alters dopamine signaling [68], which has been suggested to contribute to neuroadaptations in glutamate receptor trafficking in NAc MSNs [69]. In contrast, we did not observe an effect of withdrawal from oxycodone on dendritic spine density, consistent with our previous observation that withdrawal from oxycodone did not alter spine density in NAcC MSNs, but instead reduced spine volume and neck diameter [30]. Differential effects of opioids and psychostimulants on dendritic spines have been reported previously [62, 63]. Thus, although some synaptic consequences of opioids may be shared across drug classes, our findings suggest that adaptations in spine structure are not universal and may differ between opioids and cocaine. Here, we did not distinguish effects in D1^+^ versus D2^+^ MSNs, leaving open the possibility of cell-type-specific effects. Nonetheless, the functional synaptic adaptations observed here (e.g., changes in AMPAR/NMDAR ratio and CP-AMPAR accumulation) likely occurred in the absence of detectable changes in spine density.

### Global CA4 disruption and systemic AZD administration reduced synaptic and behavioral responses to opioid withdrawal and abstinence

Importantly, following opioid withdrawal, behavioral changes and adaptations at NAcC MSN glutamatergic synapses were prevented by CA4 disruption. However, it is not yet clear how withdrawal from opioids produces these changes or how CA4 disruption prevents them. Prior studies suggest that prolonged withdrawal from cocaine in rats decreases mGluR1 signaling and promotes CP-AMPAR trafficking to the postsynaptic membrane [21, 70, 71], presumably via reduction in PLC-mediated IP_3_-dependent Ca^2+^ release and PKC [72]. Supporting this mechanism, pharmacological activation of mGluR1 reduced synaptic CP-AMPARs in a PKC-dependent manner [21, 71]. More recently, disinhibition of retinoic acid synthesis, which can result from low intracellular Ca^2+^ [73], was implicated in homeostatically increasing synaptic CP-AMPARs following withdrawal from cocaine [23]. We observed similar withdrawal from opioid-induced synaptic adaptations in our study. We therefore speculate that withdrawal from opioids may likewise reduce mGluR1 signaling, impairing Ca²⁺ release, and homeostatically increasing CP-AMPARs into NAcC synapses. Within this framework, we previously showed that extracellular acidification drives robust Ca²⁺ increases in NAc MSNs via ASIC1A in mice via voltage-gated Ca²⁺ channels [18]. Thus, enhancing ASIC1A-mediated Ca²⁺ entry via CA4 disruption or its pharmacological inhibition with AZD may thereby oppose withdrawal-induced deficits in Ca²⁺ signaling and CP-AMPAR trafficking [18]. Additional studies are needed to rigorously test these possibilities.

Another important consideration is that the global CA4 disruption employed in these experiments leaves the precise site of CA4 action in these phenomena undefined. Our previous work demonstrated that NAcC-specific CA4 disruption prevented analogous withdrawal from cocaine-induced synaptic changes in NAcC MSNs and attenuated cocaine-evoked behavior [18], suggesting that NAcC MSNs are likely sites of CA4 action. However, the effects observed here with global deletion are also a strength because they indicate that systemically disruption or inhibition of CA4 is sufficient to blunt opioid-induced maladaptive plasticity and thus more precise interventions may be unnecessary to target CA4 therapeutically.

We previously found that disrupting or pharmacologically inhibiting CA4 increased ASIC1A-mediated synaptic currents in NAcC MSNs by lowering synaptic pH buffering capacity [17, 18]. Additionally, because loss of ASIC1A in NAcC MSNs increased sensitivity to cocaine- and opioid-induced synaptic rearrangements [17, 30], it seems reasonable to expect that the capability of CA4 disruption to enhance ASIC1A-mediated synaptic currents would produce the opposite effect and reduce sensitivity to drug-evoked synaptic changes. Consistent with the expectation that effects of CA4 disruption depend on ASIC1A, here AZD failed to affect AMPAR/NMDAR ratio in *Asic1a*^*–/–*^ mice, which exhibit increased baseline AMPAR/NMDAR ratios that are reduced in response to cocaine and opioids [17, 30].

It has been suggested that some opioid medications may modulate specific ASICs. For example, morphine has been reported to inhibit ASIC3 currents in rat DRG neurons [74]. Alternatively, oxycodone has been suggested to increase sustained ASIC3 currents in rat sensory neurons [75]. It is unlikely that these observations could explain the results in this manuscript because in the mouse brain, ASIC3 expression is low or absent [76]. It is unknown if opioids affect ASIC1A function in the brain. The synaptic effects of opioids (e.g., on AMPAR/NMDAR ratio) still occur in *Asic1a*^*–/–*^ and may be heightened [30], thus ASIC1A does not seem to be required for those rearrangements.

AZD blocks other carbonic anhydrases in addition to CA4 [40]. Thus, AZD effects might arise from effects on other CAs. The absence of AZD effects in *Car4*^*–/–*^ mice and in saline-treated *Car4*^*+/+*^ mice argues against substantial off-target effects. Still, because *Car4*^*–/–*^ mice did not exhibit oxycodone-evoked synaptic changes, their lack of AZD response does not by itself confirm CA4 as the target. Nevertheless, in *Car4*^*+/+*^ mice, AZD produced the same effects as genetically disrupting CA4. Taken together with the previous finding that AZD effects on synaptic pH buffering depend on CA4 [17], the results here support a model in which AZD reverses oxycodone-induced plasticity by inhibiting CA4.

SA remains the gold standard for modeling opioid consumption and seeking in rodents. Interestingly, CA4 disruption did not affect the acquisition of oxycodone SA, nor the total number of drug infusions. Although *Car4*^*+/+*^ control mice did not exhibit incubation of seeking behavior after extended abstinence, as recently described in rats [33], they did maintain the same amount of ALPs after 30 days of abstinence compared to baseline. However, in sharp contrast, cue-induced drug-seeking in *Car4*^*–/–*^ mice fell to half of baseline levels. Thus, CA4 disruption weakened cue-induced oxycodone seeking, which was sustained in *Car4*^*+/+*^ mice during an extended abstinence. These results echo previous effects of CA4 disruption on drug-seeking and AMPAR/NMDAR ratio following withdrawal and abstinence from cocaine SA [17, 18], suggesting that targeting CA4 may reduce seeking behavior for both drugs. Importantly, administering AZD 24 h prior to testing, at a dose used safely in humans, significantly reduced active lever presses in *Car4*^*+/+*^ mice and had no effect in *Car4*^*–/–*^ mice. These results with AZD suggest that inhibiting CA4 acutely can elicit effects similar to chronic CA4 disruption. Because the half-life of AZD in mice is ~1 h [55], the effects of AZD treatment likely lasted beyond its bioavailability.

Together, these observations strengthen the possibility that CA4 might be an effective non-opioid therapeutic target for reducing relapse in substance use disorders. Moreover, by directly modulating synaptic plasticity rather than engaging opioid receptors like current treatments [2, 77], AZD may reduce the risk of misuse or dependence, providing a safer option for the management of OUD. Additionally, supporting its safety profile, AZD’s effects appeared to be confined to drug-induced synaptic plasticity, as AZD did not alter glutamatergic transmission in drug-naïve conditions. In conclusion, these results raise the exciting possibility that AZD or similar carbonic anhydrase inhibitors, some already approved for use in humans, might be readily repurposed to reduce drug-seeking and relapse in OUD and other substance use disorders.

## Supplementary information

Statistical analysis Table S1 revised: main figures

Combined Supplementary figure legends and supplementary figures

Table S2

## Data Availability

All data supporting the conclusions of this study are available in the paper and/or the Supplementary Materials.
