# Supplementary material for: Acetazolamide inhibition of carbonic anhydrase 4 reverses opioid-induced synaptic rearrangements in nucleus accumbens and reduces drug-seeking behavior
Source: Neuropsychopharmacology. 2026 Jan 21;51(8):1402–12. doi: 10.1038/s41386-025-02319-5 (PMC13291229; doi:10.1038/s41386-025-02319-5)
Supplement: Supplementary file 1 — Statistical analysis Table S1 revised: main figures [file 41386_2025_2319_MOESM1_ESM.docx]

## Table S1.

| Figure | Dependent variables | Test | Comparison | df | F or t | P value |
| --- | --- | --- | --- | --- | --- | --- |
| 1.D | AMPAR/NMDAR ratio | 2-way ANOVA | drug × genotype interaction | 1,38 | 10.55 | 0.0024 |
|  |  | Planned contrast | Oxy vs Sal *Car4^+/+^* |  |  | <0.0001 |
|  |  | Planned contrast | Oxy vs Sal *Car4^-/-^* |  |  | 0.685 |
| 1.F | IV-curve (+50 mV) | 2-way ANOVA | drug × genotype interaction | 1,40 | 0.8833 | 0.3529 |
|  |  | 2-way ANOVA | main effect of drug | 1,40 | 1.0 | 0.3232 |
|  |  | 2-way ANOVA | main effect of genotype | 1,40 | 3.207 | 0.0809 |
|  |  | Planned contrast | Oxy WD *Car4^+/+^* vs *Car4^-/-^* |  |  | 0.0341 |
| 1.G | Rectification index | 2-way ANOVA | drug × genotype interaction | 1,40 | 6.625 | 0.0139 |
|  |  | Planned contrast | Oxy vs Sal *Car4^+/+^* |  |  | 0.0001 |
|  |  | Planned contrast | Oxy vs Sal *Car4^-/-^* |  |  | 0.8286 |
|  |  | Planned contrast | Oxy *Car4^+/+^* vs Oxy *Car4^-/-^* |  |  | <0.0001 |
| 1.I | NASPM sensitivity | 2-way ANOVA | drug × genotype interaction | 1,22 | 10.40 | 0.0039 |
|  |  | Planned contrast | Oxy vs Sal *Car4^+/+^* |  |  | <0.0001 |
|  |  | Planned contrast | Oxy vs Sal *Car4^-/-^* |  |  | 0.610 |
| 1.K | CPP score | t-test | *Car4^+/+^* vs *Car4^-/-^* | 31.31 | 2.197 | 0.0355 |
| 1.L | AMPAR/NMDAR ratio (15 mg/kg Oxy) | 2-way ANOVA | drug × genotype interaction | 1,24 | 5.987 | 0.0221 |
|  |  | Planned contrast | Oxy vs Sal *Car4^+/+^* |  |  | 0.001 |
|  |  | Planned contrast | Oxy vs Sal *Car4^-/-^* |  |  | 0.8996 |
| 2.C | AMPAR/NMDAR ratio (*Car4^+/+^*) | 2-way ANOVA | Oxy × AZD interaction | 1,37 | 4.966 | 0.032 |
|  |  | Planned contrast | Oxy WD-Veh vs Sal WD-Veh |  |  | <0.0001 |
|  |  | Planned contrast | Oxy WD-Veh vs Oxy WD-AZD |  |  | 0.0007 |
| 2.D | AMPAR/NMDAR ratio (*Car4^-/-^*) | 2-way ANOVA | Oxy × AZD interaction | 1,35 | 0.2944 | 0.5908 |
|  |  | 2-way ANOVA | main effect of Oxy | 1,35 | 1.956 | 0.1708 |
|  |  | 2-way ANOVA | main effect of AZD | 1,35 | 0.0136 | 0.9078 |
|  |  | Planned contrast | Oxy WD-Veh vs Sal WD-Veh |  |  | 0.5435 |
|  |  | Planned contrast | Oxy WD-Veh vs Oxy WD-AZD |  |  | 0.7619 |
| 2.F | Rectification index (*Car4^+/+^*) | 2-way ANOVA | Oxy × Genotype × AZD interaction | 1,41 | 6.862 | 0.0123 |
|  |  | Planned contrast | Oxy WD-Veh vs Sal WD-Veh |  |  | <0.0001 |
|  |  | Planned contrast | Oxy WD-Veh vs Oxy WD-AZD |  |  | <0.0001 |
| 2.G | Rectification index (*Car4^-/-^*) | 2-way ANOVA | Oxy × AZD interaction | 1,29 | 0.2909 | 0.5937 |
|  |  | 2-way ANOVA | main effect of Oxy | 1,29 | 0.0007 | 0.9336 |
|  |  | 2-way ANOVA | main effect of AZD | 1,29 | 1.16 | 0.2904 |
|  |  | Planned contrast | Oxy WD-Veh vs Sal WD-Veh |  |  | 0.7411 |
|  |  | Planned contrast | Oxy WD-Veh vs Oxy WD-AZD |  |  | 0.2279 |
| 2.I | NASPM sensitivity (*Car4^+/+^*) | 2-way ANOVA | Oxy × AZD interaction | 1,23 | 18.38 | 0.0003 |
|  |  | Planned contrast | Oxy WD-Veh vs Sal WD-Veh |  |  | <0.0001 |
|  |  | Planned contrast | Oxy WD-Veh vs Oxy WD-AZD |  |  | <0.0001 |
| 2.J | NASPM sensitivity  (*Car4^-/-^*) | 2-way ANOVA | Oxy × AZD interaction | 1,19 | 0.6252 | 0.4389 |
|  |  | 2-way ANOVA | main effect of Oxy | 1,19 | 0.06 | 0.8092 |
|  |  | 2-way ANOVA | main effect of AZD | 1,19 | 0.1131 | 0.7403 |
|  |  | Planned contrast | Oxy WD-Veh vs Sal WD-Veh |  |  | 0.6969 |
|  |  | Planned contrast | Oxy WD-Veh vs Oxy WD-AZD |  |  | 0.4458 |
| 3.C | AMPAR/NMDAR ratio-Heroin (H) | 2-way ANOVA | H × AZD interaction | 1,32 | 6.156 | 0.0185 |
|  |  | Planned contrast | Sal-Veh vs H-Veh |  |  | 0.0006 |
|  |  | Planned contrast | H-Veh vs H-AZD |  |  | 0.0043 |
|  |  | Planned contrast | H-Veh vs Sal-AZD |  |  | 0.0034 |
| 3.E | AMPAR/NMDAR ratio-Morphine (Mor) | 2-way ANOVA | Mor × AZD interaction | 1,26 | 2.452 | 0.1295 |
|  |  | 2-way ANOVA | main effect of Morphine | 1,26 | 8.088 | 0.0086 |
|  |  | 2-way ANOVA | main effect of AZD | 1,26 | 7.179 | 0.0126 |
|  |  | Planned contrast | Sal-Veh vs Mor-Veh |  |  | 0.0025 |
|  |  | Planned contrast | Mor-Veh vs  Mor-AZD |  |  | 0.0074 |
|  |  | Planned contrast | Mor-Veh vs Sal-AZD |  |  | 0.0005 |
| 3.H | AZD effect on  AMPAR/NMDAR ratio (*Asic1a^-/-^)* | 2-way ANOVA | Oxy × AZD interaction | 1,30 | 0.2836 | 0.5983 |
|  |  | 2-way ANOVA | Oxy main effect | 1,30 | 45.97 | <0.0001 |
|  |  | 2-way ANOVA | AZD main effect | 1,30 | 0.002977 | 0.9569 |
|  |  | Planned contrast | Sal-Veh vs Sal-AZD |  |  | 0.6895 |
|  |  | Planned contrast | Oxy-Veh vs Oxy-AZD |  |  | 0.73 |
| 4.D | AMPAR/NMDAR ratio (D1^+^ MSNs) | 2-way ANOVA | Oxy × AZD interaction | 1,19 | 8.578 | 0.0086 |
|  |  | Planned contrast | Oxy-Veh vs Sal-Veh |  |  | 0.0005 |
|  |  | Planned contrast | Oxy-Veh vs Oxy-AZD |  |  | 0.0006 |
|  |  | Planned contrast | Oxy-Veh vs Sal-AZD |  |  | 0.001 |
| 4.F | AMPAR/NMDAR ratio (non-D1^+^ MSNs) | 2-way ANOVA | Oxy × AZD interaction | 1,19 | 0.157 | 0.9017 |
|  |  | 2-way ANOVA | Oxy main effect | 1,19 | 0.0838 | 0.7754 |
|  |  | 2-way ANOVA | AZD main effect | 1,19 | 1.609 | 0.220 |
|  |  | Planned contrast | Oxy-Veh vs Sal-Veh |  |  | 0.7688 |
|  |  | Planned contrast | Oxy-Veh vs Oxy-AZD |  |  | 0.3047 |
|  |  | Planned contrast | Oxy-Veh vs Sal-AZD |  |  | 0.2765 |
| 5.B | Active lever presses | 2-way ANOVA | Genotype main effect | 1,30 | 0.2533 | 0.6187 |
|  | Inactive lever presses | 2-way ANOVA | Genotype main effect | 1,30 | 0.8632 | 0.3602 |
| 5.C | Infusions | 2-way ANOVA | Session × Genotype interaction | 1,200 | 1.152 | 0.3278 |
|  |  | 2-way ANOVA | Genotype main effect | 1,20 | 0.4102 | 0.5291 |
| 5.D | Active lever presses | 3-way ANOVA with repeated measures | Genotype × AZD × time  interaction | 1,35 | 2.000 | 0.1661 |
|  |  | 3-way ANOVA with repeated measures | Genotype × time interaction | 1,35 | 4.733 | 0.0364 |
|  | Active lever presses (*Car4^+/+^*, Veh) | Paired t-test | Day 11 vs Day 41 | 8 | 0.4473 | 0.6665 |
|  | Active lever presses (*Car4^+/+^*, AZD) | Paired t-test | Day 11 vs Day 41 | 9 | 3.333 | 0.0088 |
|  | Active lever presses (*Car4^-/-^*, Veh) | Paired t-test | Day 11 vs Day 41 | 9 | 2.529 | 0.0323 |
|  | Active lever presses (*Car4^-/-^*, AZD) | Paired t-test | Day 11 vs Day 41 | 9 | 4.398 | 0.0017 |
| 5.E | Normalized Day 41 active presses | 2-way ANOVA | Genotype × AZD interaction | 1,35 | 4.252 | 0.0467 |
|  |  | Planned contrast | *Car4^+/+^* Veh vs *Car4^+/+^* AZD |  |  | 0.0143 |
|  |  | Planned contrast | *Car4^+/+^* Veh vs *Car4^-/-^* Veh |  |  | 0.0190 |
|  |  | Planned contrast | *Car4^+/+^* Veh vs *Car4^-/-^* AZD |  |  | 0.0150 |
| 5.F | Inactive lever presses | 3-way ANOVA | Genotype × AZD × time interaction | 1,35 | 0.0006325 | 0.9801 |
|  | Inactive lever presses (*Car4^+/+^*, Veh) | Paired t-test | Day 11 vs Day 41 | 8 | 0.1313 | 0.8987 |
|  | Inactive lever presses (*Car4^+/+^*, AZD) | Paired t-test | Day 11 vs Day 41 | 9 | 1.296 | 0.2272 |
|  | Inactive lever presses (*Car4^-/-^*, Veh) | Paired t-test | Day 11 vs Day 41 | 9 | 1.513 | 0.1647 |
|  | Inactive lever presses (*Car4^-/-^*, AZD) | Paired t-test | Day 11 vs Day 41 | 9 | 3.591 | 0.0058 |
| 5.H | AMPAR/NMDAR ratio (post-SA) | 2-way ANOVA | Genotype × AZD interaction | 1,51 | 5.126 | 0.0278 |
|  |  | Planned contrast | *Car4^+/+^* Veh vs *Car4^+/+^* AZD |  |  | 0.0011 |
|  |  | Planned contrast | *Car4^+/+^* Veh vs *Car4^-/-^*Veh |  |  | 0.0006 |
|  |  | Planned contrast | *Car4^+/+^* Veh vs *Car4^-/-^*AZD |  |  | 0.0045 |

**Table S1. Summary of statistical analyses for the main figures.**
This table reports all dependent variables, statistical tests, degrees of freedom, F/t values, and p values. Main effects, interactions, and planned post hoc comparisons are included where appropriate.
