# Supplementary material for: Acetazolamide inhibition of carbonic anhydrase 4 reverses opioid-induced synaptic rearrangements in nucleus accumbens and reduces drug-seeking behavior
Source: Neuropsychopharmacology. 2026 Jan 21;51(8):1402–12. doi: 10.1038/s41386-025-02319-5 (PMC13291229; doi:10.1038/s41386-025-02319-5)
Supplement: Supplementary file 2 — Combined Supplementary figure legends and supplementary figures [file 41386_2025_2319_MOESM2_ESM.pdf]

Supplementary Figure Legends:

Fig. S1. Effects of oxycodone withdrawal and CA4 are retained when quantifying AMPAR/NMDAR ratio using peak NMDAR amplitude instead of sustained NMDAR current.

Car4$^{+/+}$ and *Car4*$^{-/-}$ mice were administered 15 mg/kg ip oxycodone vs. saline for 5 days, followed by 10 days of withdrawal, then AMPAR/NMDAR was assessed. Here peak NMDAR amplitude was used to quantify AMPAR/NMDAR ratio instead of NMDAR amplitude 60 ms after EPSC onset as in other figures. With this alternative approach a significant oxycodone by genotype interaction was still observed (n = 6-8 neurons from 3 mice/group).

Fig. S2. AMPAR/NMDAR ratio results separated by sex, genotype, and AZD treatment.

Although there were strong effects of oxycodone withdrawal, *Car4* genotype, and AZD, there were no significant interactions between these factors and sex (n = 5 to 6 neurons from 2 to 3 mice/group).

Fig. S3. Oxycodone withdrawal-induced increase in AMPAR/NMDAR ratio in NAcC MSNs required an extended abstinence period (>24 hrs).

(A) Oxycodone (oxy) dosing paradigms administered to *Car4*$^{+/+}$ mice to assess effects on AMPAR/NMDAR ratio: (i) 5 injections and 10 days of withdrawal (ii) 5 days of injections and 24 hrs withdrawal (iii) a single injection and 24 hrs withdrawal.

(B) AMPAR/NMDAR ratio increased in group (i), but not in groups (ii) or (iii) (n = 7-13 neurons from 3 to 4 mice/group).

Fig. S4. *In vitro* and *in vivo* administration of the CA inhibitor AZD reversed effects of opioid withdrawal on AMPAR/NMDAR ratio.

(A) Experimental timeline: oxycodone (3mg/kg, i.p.) or saline (i.p.) was administered each day for 5 days, followed by 5 days of withdrawal. Brain slices were prepared, and AZD 100µM or vehicle (ACSF) was added to the recording chamber for 60 minutes before testing.

(B) The AMPAR/NMDAR ratio increased after oxycodone withdrawal (Oxy) in *Car4*$^{+/+}$ mice. Applying AZD to the recording chamber reversed values to control levels (n = 7-12 neurons from 3-4 mice/group).

(C) Experimental timeline: oxycodone (3 mg/kg) vs. saline (i.p, 5 days), followed by 5 days of withdrawal. 3 hrs later, AZD (30 mg/kg) vs. vehicle was administered *in vivo*, and slices were harvested for electrophysiological recording.

(D) AZD treatment reversed the oxycodone withdrawal-induced increase in AMPAR/NMDAR ratio (n = 7-8 neurons from 3 mice/group).

(E) Experimental timeline: morphine (10mg/kg), or saline (i.p.) was administered for 5 days, followed by 5 days of withdrawal. AZD (30/mg/kg) or vehicle was administered by i.p. injection in vivo, and 3 hrs later, slices were harvested for electrophysiological recording.

(F) *In vivo* administration of AZD reversed AMPAR/NMDAR ratios following morphine abstinence to control levels (n = 6-9 neurons from 3 mice/group).

Fig. S5. Low-dose heroin (2mg/kg) followed by withdrawal did not affect AMPAR/NMDAR ratio in NAcC MSNs of *Car4*$^{+/+}$ mice

(A) Experimental timeline of heroin treatment and electrophysiology

(B) Abstinence from low-dose heroin did not change the AMPAR/NMDAR ratio and AZD had no effect (n = 9-12 neurons from 4 mice/group).

Fig. S6. AMPAR/NMDAR ratio was increased in NAcC MSNs of *Car4*$^{+/+}$ mice after 5 days of morphine withdrawal, and AZD normalized it to control levels.

(A) Experimental paradigm of morphine treatment and electrophysiology

(B) AMPAR/NMDAR ratio was increased after 5 days of morphine withdrawal and was normalized by AZD (n = 7-13 neurons from 4 mice/group).

Fig. S7. Oxycodone withdrawal and AZD had no effect on dendritic spine densities in NAcC MSNs

(A) Experimental timeline: oxycodone (3mg/kg, i.p.) or saline (i.p.) was administered in the home cage each day for 5 days, followed by 10 days of withdrawal, after which DiI labeling was performed.

(B) Representative images (projected z-stack) of dendritic spines in NAcC MSNs from *Car4*$^{+/+}$ mice withdrawn from oxycodone (Oxy) vs saline (Sal) and treated with AZD vs. vehicle.

(C) Total spine density (average # spines/um per neuron) was unchanged by oxycodone withdrawal and AZD (n = 9 - 16 neurons per group from 3 - 4 mice and 2 - 4 dendritic segments (50 - 60 μm) averaged per neuron.

(D) Stubby spine density was unchanged by oxycodone withdrawal and AZD (Oxy by AZD interaction.

(E) Thin spine density was unchanged by oxycodone withdrawal and AZD.

(F) Mushroom spine density was unchanged by oxycodone withdrawal and AZD.

Fig S1.

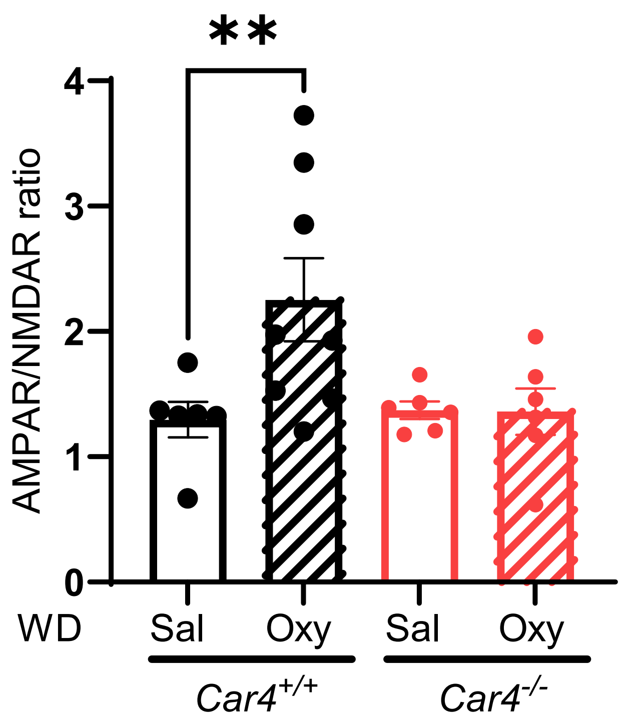

Fig S2.

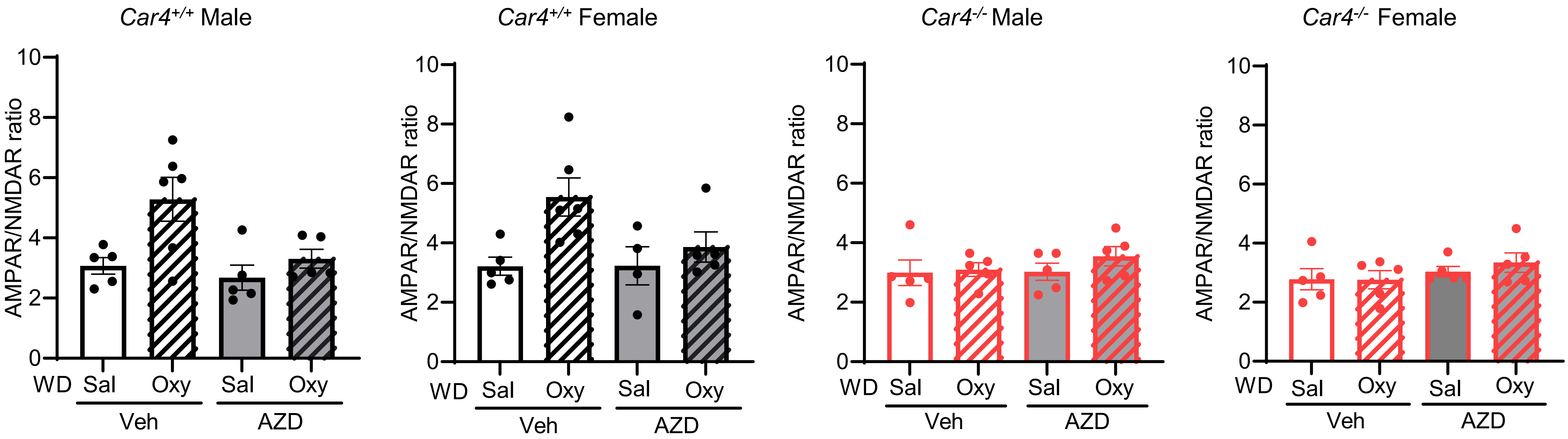
Car4+/+ Male
Car4+/+ Female
Car4-/- Male
Car4-/- Female
AMPAR/NMDAR ratio
0
2
4
6
8
10
WD
Sal
Oxy
Sal
Oxy
Veh
AZD

Fig S3.

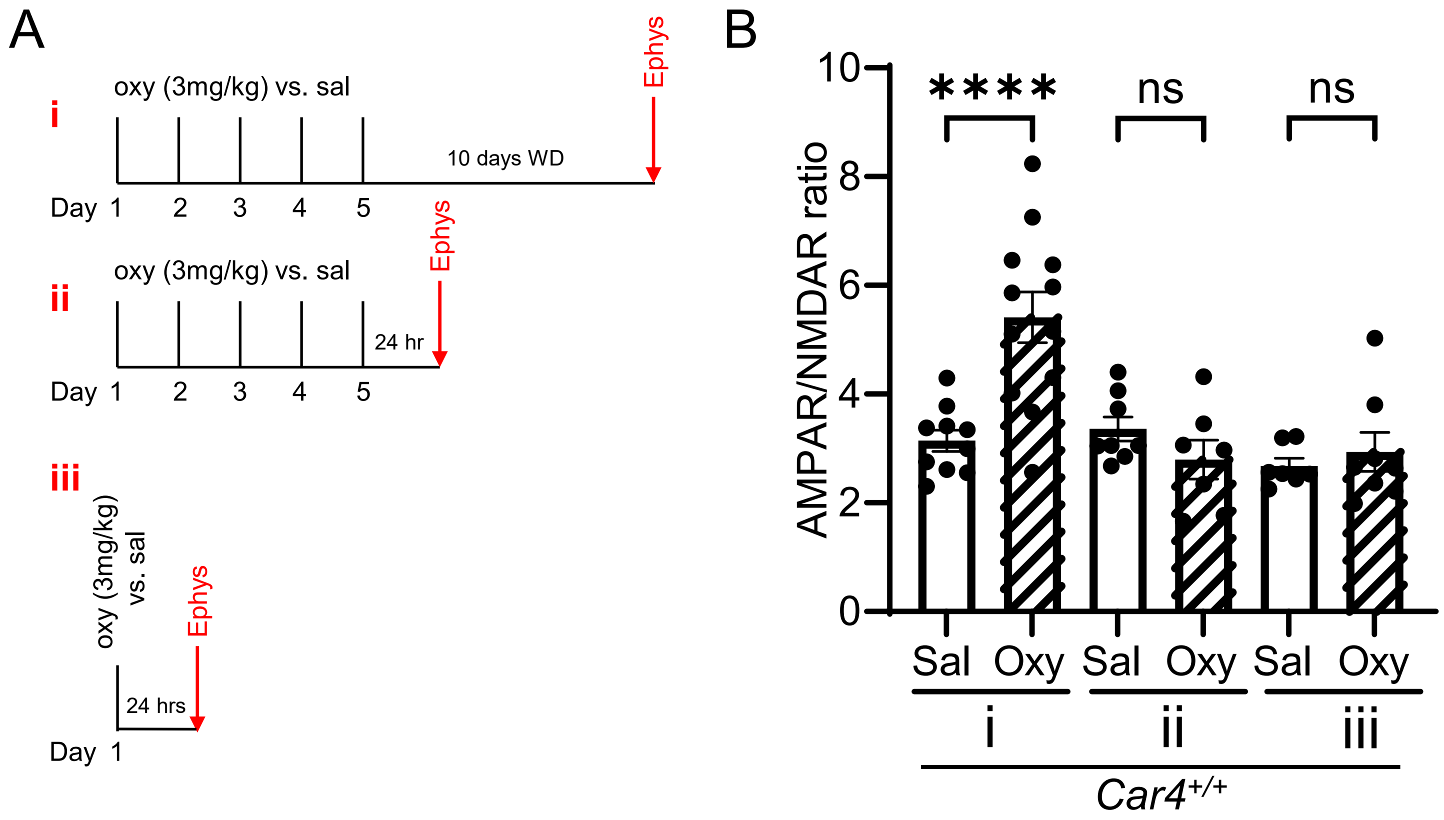

A
i
oxy (3mg/kg) vs. sal
10 days WD
Ephys
Day 1 2 3 4 5
ii
oxy (3mg/kg) vs. sal
24 hr
Ephys
Day 1 2 3 4 5
iii
oxy (3mg/kg) vs. sal
24 hrs
Ephys
Day 1
B
AMPAR/NMDAR ratio
0 2 4 6 8 10
****
ns
ns
Sal Oxy Sal Oxy Sal Oxy
i ii iii
Car4+/+

Fig S4.

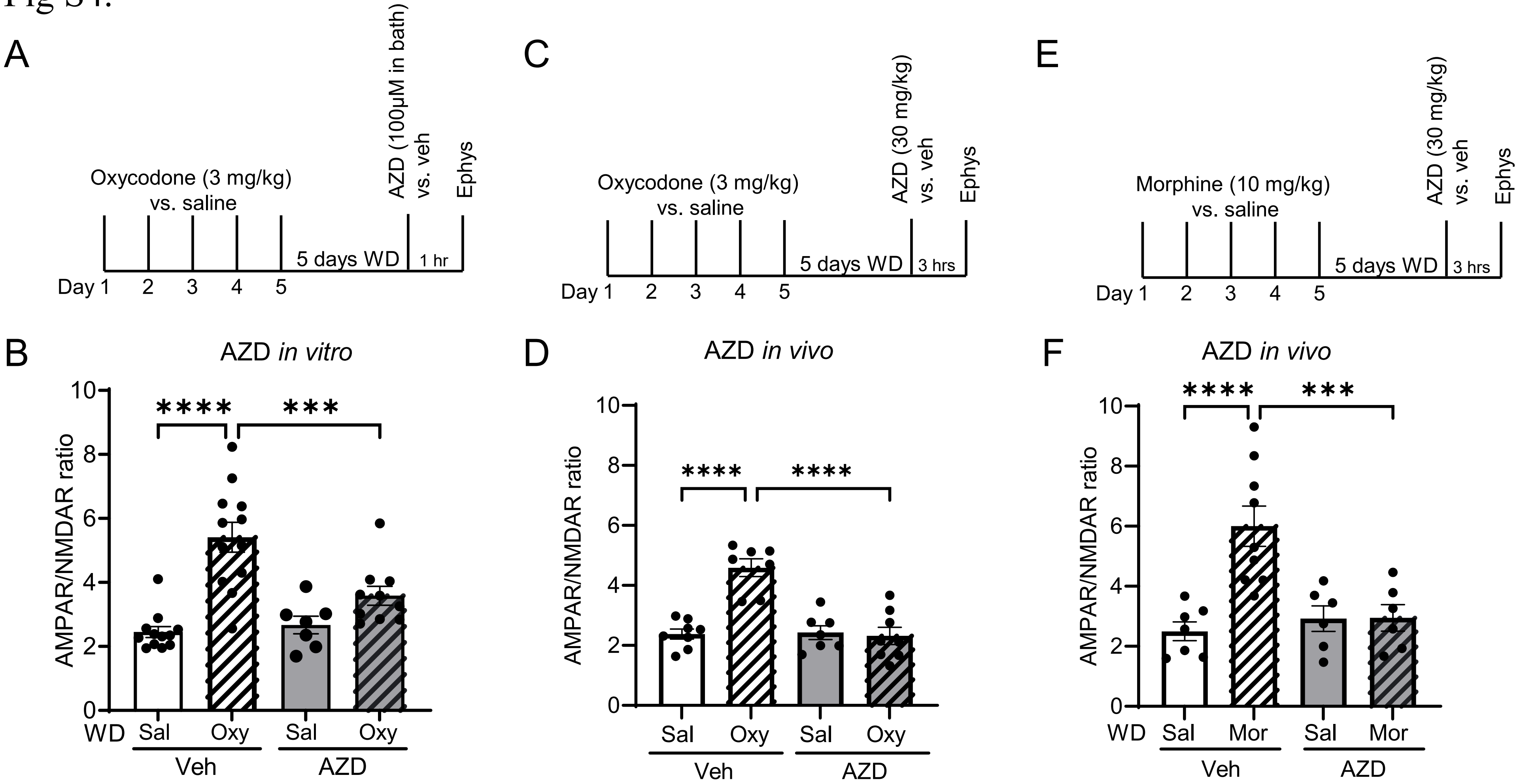
A
Oxycodone (3 mg/kg)
vs. saline
AZD (100μM in bath)
vs. veh
Ephys
5 days WD
1 hr
Day 1
2
3
4
5
B
AZD *in vitro*
AMPAR/NMDAR ratio
10
8
6
4
2
0
****
***
WD
Sal
Oxy
Sal
Oxy
Veh
AZD
C
Oxycodone (3 mg/kg)
vs. saline
AZD (30 mg/kg)
vs. veh
Ephys
5 days WD
3 hrs
Day 1
2
3
4
5
D
AZD *in vivo*
AMPAR/NMDAR ratio
10
8
6
4
2
0
****
****
Sal
Oxy
Sal
Oxy
Veh
AZD
E
Morphine (10 mg/kg)
vs. saline
AZD (30 mg/kg)
vs. veh
Ephys
5 days WD
3 hrs
Day 1
2
3
4
5
F
AZD *in vivo*
AMPAR/NMDAR ratio
10
8
6
4
2
0
****
***
WD
Sal
Mor
Sal
Mor
Veh
AZD

Fig S5.

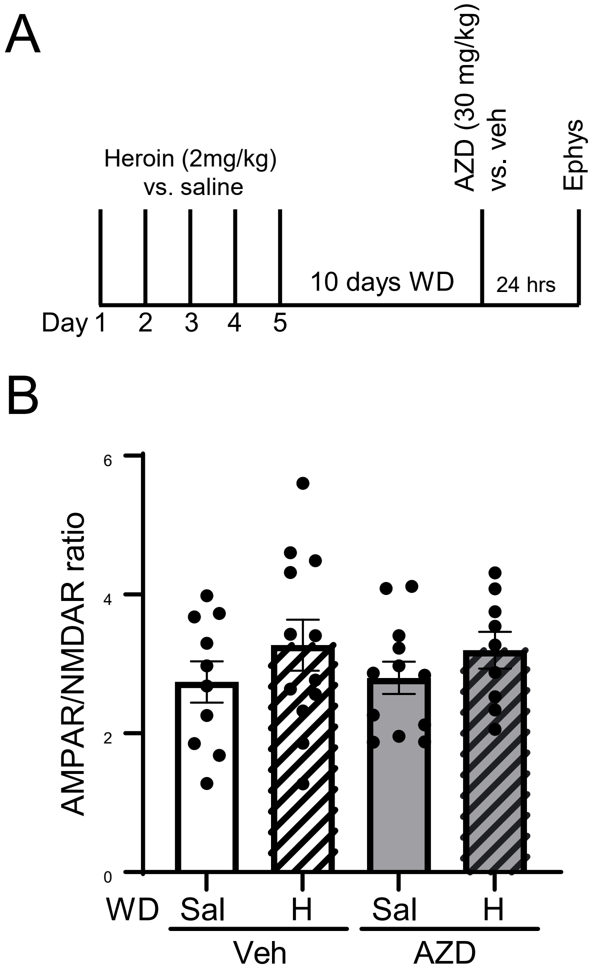
A
Heroin (2mg/kg)
vs. saline
AZD (30 mg/kg)
vs. veh
Ephys
10 days WD
24 hrs
Day 1 2 3 4 5
B
AMPAR/NMDAR ratio
6
4
2
0
WD Sal H Sal H
Veh
AZD

Fig S6.

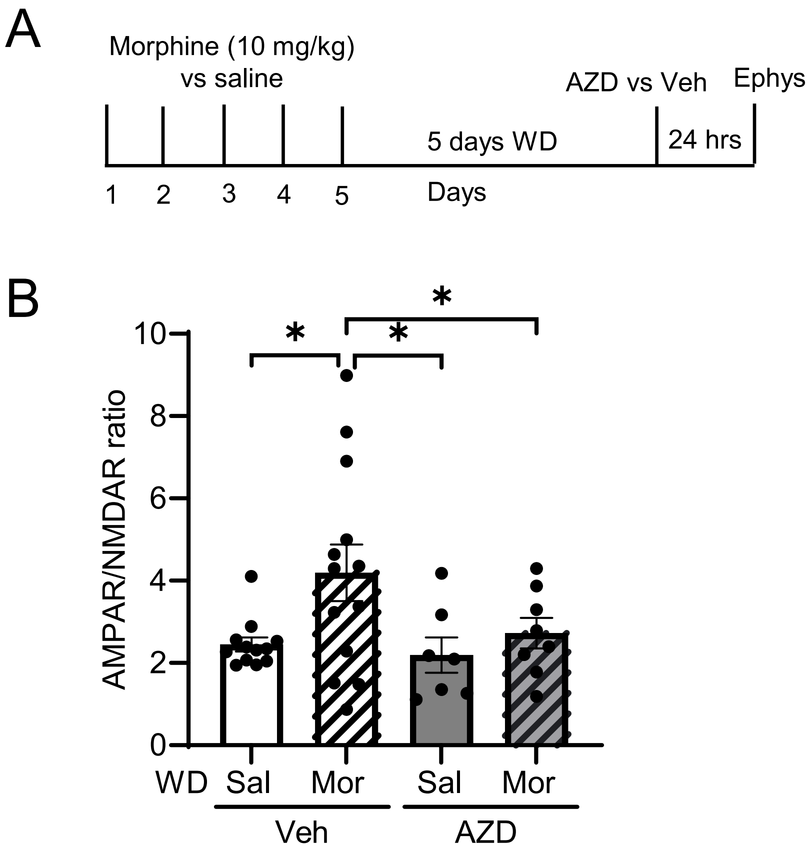
A
Morphine (10 mg/kg)
vs saline
AZD vs Veh
Ephys
5 days WD
24 hrs
1
2
3
4
5
Days
B
*
*
*
10
8
6
4
2
0
AMPAR/NMDAR ratio
WD
Sal
Mor
Sal
Mor
Veh
AZD

Fig S7.

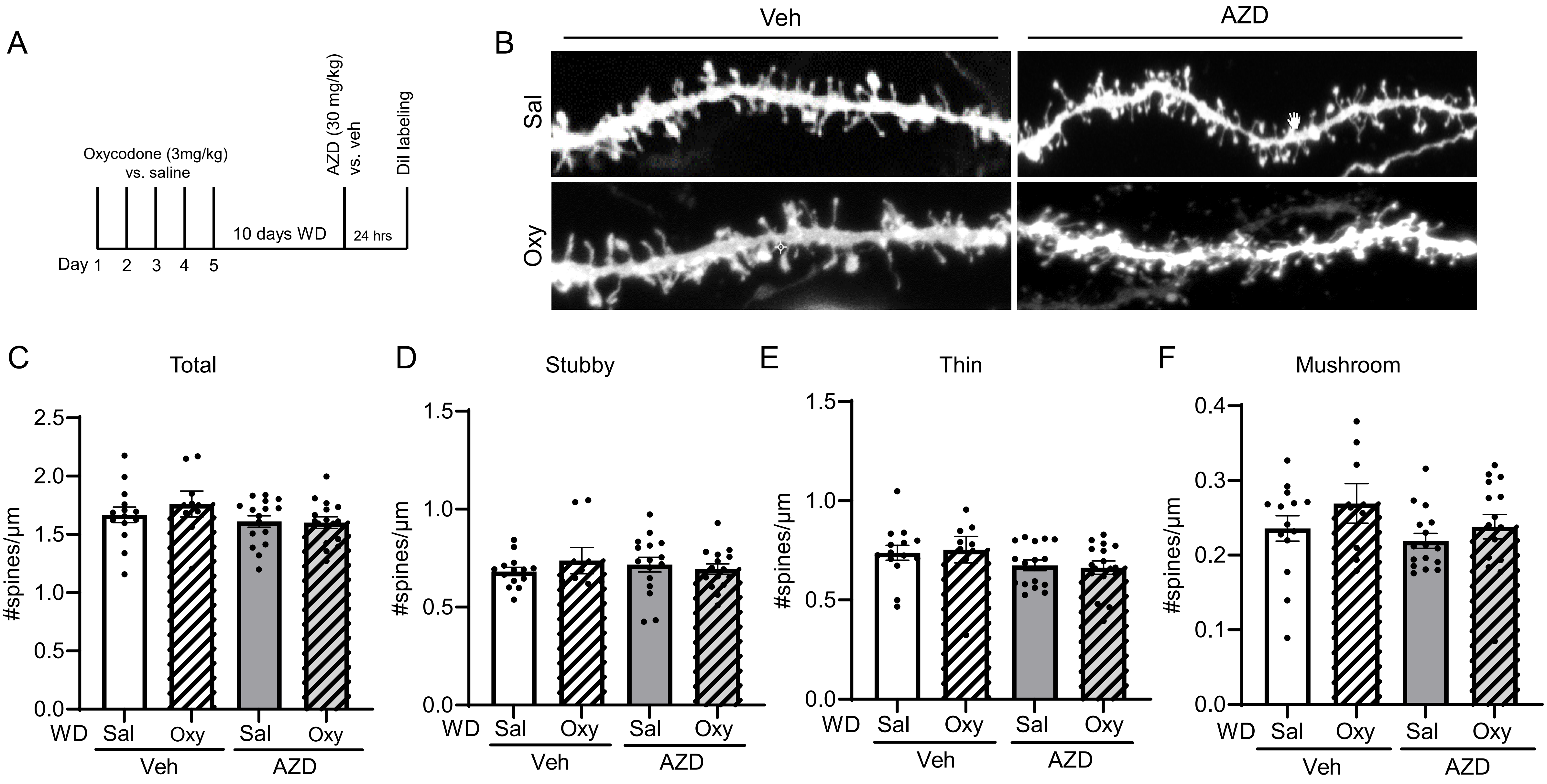

A
Oxycodone (3mg/kg)
vs. saline
AZD (30 mg/kg)
vs. veh
DiI labeling
10 days WD
24 hrs
Day 1 2 3 4 5
B
Veh
AZD
Sal
Oxy
C
Total
#spines/μm
2.5
2.0
1.5
1.0
0.5
0.0
WD Sal Oxy Sal Oxy
Veh AZD
D
Stubby
#spines/μm
1.5
1.0
0.5
0.0
WD Sal Oxy Sal Oxy
Veh AZD
E
Thin
#spines/μm
1.5
1.0
0.5
0.0
WD Sal Oxy Sal Oxy
Veh AZD
F
Mushroom
#spines/μm
0.4
0.3
0.2
0.1
0.0
WD Sal Oxy Sal Oxy
Veh AZD
