# Supplementary material for: Acetazolamide inhibition of carbonic anhydrase 4 reverses opioid-induced synaptic rearrangements in nucleus accumbens and reduces drug-seeking behavior
Source: Neuropsychopharmacology. 2026 Jan 21;51(8):1402–12. doi: 10.1038/s41386-025-02319-5 (PMC13291229; doi:10.1038/s41386-025-02319-5)
Supplement: Supplementary file 3 — Table S2 [file 41386_2025_2319_MOESM3_ESM.docx]

## Table S2.

| **Figure** | **Dependent variables** | **Test** | **Comparison** | **df** | **F or t** | **P value** |
| --- | --- | --- | --- | --- | --- | --- |
| S1 | AMPAR/NMDAR ratio using peak amplitude of NMDAR component | 2-way ANOVA | drug × genotype interaction | 1,24 | 4.819 | 0.0381 |
|  |  | Planned contrast | Oxy vs Sal *Car4^+/+^* |  |  | 0.0029 |
| S2 | AMPAR/NMDAR ratio | 2-way ANOVA | Veh-treated *Car4^+/+^* grps, Oxy vs. Sal, M vs. F,  Oxy × Sex interaction | 1,18 | 0.011 | 0.9176 |
|  |  | 2-way ANOVA | Veh-treated *Car4^+/+^* grps, Oxy vs. Sal, M vs. F,  Oxy main effect | 1,18 | 16.03 | 0.0008 |
|  |  | 2-way ANOVA | Veh-treated *Car4^+/+^* grps, Oxy vs. Sal, M vs. F, Sex main effect | 1,18 | 0.1292 | 0.7235 |
|  |  | 2-way ANOVA | Veh-treated Oxy grps, *Car4^+/+^* vs. *Car4^-/-^*, M vs. F, Genotype × Sex interaction | 1,18 | 0.2851 | 0.5999 |
|  |  | 2-way ANOVA | Veh-treated Oxy grps, *Car4^+/+^* vs. *Car4^-/-^*, M vs. F, Genotype main effect | 1,18 | 19.41 | 0.0003 |
|  |  | 2-way ANOVA | Veh-treated oxy grps, *Car4^+/+^* vs. *Car4^-/-^*, M vs. F, Sex main effect | 1,18 | 0.0045 | 0.9475 |
|  |  | 2-way ANOVA | Oxy, *Car4^+/+^* grps, AZD vs. veh, M vs. F, AZD × Sex interaction | 1,18 | 0.0607 | 0.8082 |
|  |  | 2-way ANOVA | Oxy, *Car4^+/+^* grps, AZD vs. veh, M vs. F, AZD main effect | 1,18 | 9.293 | 0.0069 |
|  |  | 2-way ANOVA | Oxy, *Car4^+/+^* grps, AZD vs. veh, M vs. F, Sex main effect | 1,18 | 0.4699 | 0.5018 |
| S3.B(i) | AMPAR/NMDAR ratio | t-test | Sal vs Oxy (5 inj, 10 d WD) | 20 | 4.203 | 0.0004 |
| S3.B(ii) | AMPAR/NMDAR ratio | t-test | Sal vs Oxy (5 inj, 24 h WD) | 12 | 0.3123 | 0.7602 |
| S3.B(iii) | AMPAR/NMDAR ratio | t-test | Sal vs Oxy (1 inj, 24 h WD) | 14 | 1.006 | 0.3318 |
| S4.B | AMPAR/NMDAR ratio (in vitro AZD) | 2-way ANOVA | Oxy × AZD interaction | 1,37 | 8.729 | 0.0054 |
|  |  | Planned contrast | Oxy-Veh vs Sal-Veh |  |  | <0.0001 |
|  |  | Planned contrast | Oxy-Veh vs Oxy-AZD |  |  | 0.0003 |
|  |  | Planned contrast | Oxy-Veh vs Sal-AZD |  |  | <0.0001 |
| S4.D | AMPAR/NMDAR ratio (in vivo Oxy) | 2-way ANOVA | Oxy × AZD interaction | 1,26 | 21.69 | <0.0001 |
|  |  | Planned contrast | Oxy-Veh vs Sal-Veh |  |  | <0.0001 |
|  |  | Planned contrast | Oxy-Veh vs Oxy-AZD |  |  | <0.0001 |
| S4.F | AMPAR/NMDAR ratio (in vivo Morphine (Mor) | 2-way ANOVA | Mor × AZD interaction | 1,24 | 10.34 | 0.0196 |
|  |  | Planned contrast | Mor-Veh vs Sal-Veh |  |  | <0.0001 |
|  |  | Planned contrast | Mor-Veh vs Mor-AZD |  |  | 0.0004 |
| S5.B | AMPAR/NMDAR ratio (low-dose heroin(H) | 2-way ANOVA | H × AZD interaction | 1,39 | 0.0492 | 0.8257 |
|  |  | 2-way ANOVA | H main effect | 1,39 | 2.324 | 0.1355 |
|  |  | 2-way ANOVA | AZD main effect | 1,39 | 0.0007 | 0.9794 |
|  |  | Planned contrast | Sal WD-Veh vs H WD-Veh |  |  | 0.2175 |
|  |  | Planned contrast | H WD-Veh vs H WD-AZD |  |  | 0.8638 |
|  |  | Planned contrast | H WD-Veh vs Sal WD-AZD |  |  | 0.2502 |
| S6.B | AMPAR/NMDAR ratio (Morphine (Mor) WD) | 2-way ANOVA | Mor × AZD interaction | 1,36 | 1.327 | 0.2569 |
|  |  | 2-way ANOVA | Mor main effect | 1,36 | 4.659 | 0.0369 |
|  |  | 2-way ANOVA | AZD main effect | 1,36 | 2.697 | 0.1093 |
|  |  | Planned contrast | Sal WD-Veh vs Mor WD-Veh |  |  | 0.0102 |
|  |  | Planned contrast | Mor WD-Veh vs Mor WD-AZD |  |  | 0.0493 |
|  |  | Planned contrast | Sal WD-AZD vs Mor WD-Veh |  |  | 0.0116 |
| S7.C | Total spine density | 2-way ANOVA | Oxy × AZD interaction | 1,49 | 0.6042 | 0.4407 |
|  |  | 2-way ANOVA | Oxy main effect | 1,49 | 0.3975 | 0.5313 |
|  |  | 2-way ANOVA | AZD main effect | 1,49 | 2.694 | 0.1071 |
|  |  | Planned contrast | Oxy-Veh vs Sal-Veh |  |  | 0.3692 |
|  |  | Planned contrast | Oxy-Veh vs Oxy-AZD |  |  | 0.1214 |
|  |  | Planned contrast | Sal-Veh vs Sal-AZD |  |  | 0.5057 |
| S7.D | Stubby spine density | 2-way ANOVA | Oxy × AZD interaction | 1,49 | 1.121 | 0.2949 |
|  |  | 2-way ANOVA | Oxy main effect | 1,49 | 0.2006 | 0.6562 |
|  |  | 2-way ANOVA | AZD main effect | 1,49 | 0.0138 | 0.9071 |
|  |  | Planned contrast | Oxy-Veh vs Sal-Veh |  |  | 0.3368 |
|  |  | Planned contrast | Oxy-Veh vs Oxy-AZD |  |  | 0.4471 |
|  |  | Planned contrast | Sal-Veh vs Sal-AZD |  |  | 0.4668 |
| S7.E | Thin spine density | 2-way ANOVA | Oxy × AZD interaction | 1,49 | 0.1144 | 0.7367 |
|  |  | 2-way ANOVA | Oxy main effect | 1,49 | 0.0033 | 0.9542 |
|  |  | 2-way ANOVA | AZD main effect | 1,49 | 3.799 | 0.0570 |
|  |  | Planned contrast | Oxy-Veh vs Sal-Veh |  |  | 0.7999 |
|  |  | Planned contrast | Oxy-Veh vs Oxy-AZD |  |  | 0.1425 |
|  |  | Planned contrast | Sal-Veh vs Sal-AZD |  |  | 0.2151 |
| S7.F | Mushroom spine density | 2-way ANOVA | Oxy × AZD interaction | 1,49 | 0.1803 | 0.6729 |
|  |  | 2-way ANOVA | Oxy main effect | 1,49 | 2.390 | 0.1286 |
|  |  | 2-way ANOVA | AZD main effect | 1,49 | 1.999 | 0.1638 |
|  |  | Planned contrast | Oxy-Veh vs Sal-Veh |  |  | 0.2106 |
|  |  | Planned contrast | Oxy-Veh vs Oxy-AZD |  |  | 0.2367 |
|  |  | Planned contrast | Sal-Veh vs Sal-AZD |  |  | 0.4444 |

**Table S2. Summary of statistical analyses for supplementary figures.**
This table reports all dependent variables, statistical tests, degrees of freedom, F/t values, and p values. Main effects, interactions, and planned contrasts are presented where appropriate. Sex is indicated as M = male and F = female.
